# Amplitude rise time sensitivity in children with and without dyslexia: differential task effects and longitudinal relations to phonology and literacy

**DOI:** 10.3389/fpsyg.2024.1245589

**Published:** 2024-07-23

**Authors:** Sheila Flanagan, Angela M. Wilson, Fiona C. Gabrielczyk, Annabel MacFarlane, Kanad N. Mandke, Usha Goswami

**Affiliations:** Centre for Neuroscience in Education, Department of Psychology, University of Cambridge, Cambridge, United Kingdom

**Keywords:** amplitude rise time, frequency rise, developmental dyslexia, phonology, literacy, speech, spectrotemporal, rhythm

## Abstract

The speech amplitude envelope carries important acoustic information required for speech intelligibility and contains sensory cues (amplitude rise times, ARTs) that play a key role in both sensory rhythm perception and neural speech encoding. Individual differences in children’s sensitivity to ARTs have been related to the development of children’s phonological processing skills across languages by the Temporal Sampling theory. Impaired processing of ARTs also characterises children with dyslexia. However, different ART tasks have been employed in different studies, in different languages, and at different ages. Here, we compare the sensitivity of three frequently used ART tasks (based on synthetic syllables, sine tones, and speech-shaped noise) in a longitudinal study of English-speaking children with and without dyslexia. Children’s ability to discriminate rising frequency, duration, and intensity was also tested. ART discrimination in all 3 tasks was significantly inter-related, but different relations to phonology and literacy were found for different ART tasks at different ages. In particular, the often-used sine tone and speech-shaped noise ART tasks showed greater sensitivity in older children, while the synthetic syllable task (/ba/ rise) showed greater sensitivity in younger children. Sensitivity to rising frequency was also related to phonology and literacy across ages. The data are interpreted with respect to the Temporal Sampling theory of developmental dyslexia.

## 1 Introduction

A significant association between children’s rhythmic awareness and their phonological development has long been recognised, and rhythmic skills are also related to individual differences in learning language and learning to read (Fiveash et al., 2021, for review). The sensory perception of rhythm is intimately related to amplitude rise time discrimination, as described by the Temporal Sampling (TS) theory (Goswami, 2011). Acoustic rhythmic structure is carried in the amplitude modulation (AM) of the speech amplitude envelope (Greenberg et al., 2006). The amplitude envelope is the overall energy profile, which in speech is dominated by slow-varying, i.e., low-frequency modulations, for example, at the ‘prosodic rate’ of ~2 Hz AM and the ‘syllable rate’ of ~5 Hz AM (Greenberg et al., 2003). Amplitude envelope rise times are sensory cues to these (and other) different temporal rates of amplitude modulation in speech. TS theory (Goswami, 2011) posits that the primary neural deficit in dyslexia relates to impaired phase locking to the relevant low-frequency AMs (<10 Hz) in the amplitude envelope by auditory oscillatory networks operating at these low frequencies. Neural oscillatory phase locking is an automatic process triggered by ART discrimination (Gross et al., 2013; Doelling et al., 2014).

These sensory processes are thought to be related to the phonological development in the TS theory because the onsets of successive syllable- and stressed syllable-related modulations in the amplitude envelope and their amplitude rise times (ARTs) are critical linguistic perceptual events. ARTs vary with the phonetic properties of the syllable (e.g., whether it starts with a plosive like /b/or a glide like/w/) and are more salient perceptually when a syllable is stressed or acoustically strong (Goswami and Leong, 2013). ART discrimination is frequently impaired in children with developmental dyslexia, who also show impairments in perceiving and producing rhythm (Goswami, 2022, for review). Consequently, it has been suggested that the phonological difficulties that characterise children with dyslexia across languages may relate initially to acoustic problems at the syllabic and supra-syllabic levels of phonology that are related to speech rhythm and not to acoustic problems at the letter-sound correspondence (phonetic or phonemic) level (Goswami et al., 2002).

To first investigate this rhythmic hypothesis (later described by TS theory; Goswami, 2011), a beat-based perceptual categorisation task based on an amplitude-modulated sine tone was developed (Goswami et al., 2002). Perceptual sensitivity in this task (i.e., the degree of ART change needed to hear a rhythmic “beat”) was found to be significantly impaired in children with developmental dyslexia and significantly enhanced in precocious readers. In the sample of 101 children, individual differences in beat detection were related to reading and spelling development and phonological processing, including measures of phonological awareness (onset-rime), phonological short-term memory (PSTM), and rapid automatised naming (RAN). This was theoretically interesting, as these 3 domains of phonological processing had been identified in a seminal review of reading acquisition that suggested a causal relationship between phonological development and reading development (Wagner and Torgesen, 1987; see also Stanovich, 1984).

Individual differences between children in both RAN and phonological awareness are known to be significant predictors of progress in reading across languages, with phonological short-term memory (PSTM) showing a more mixed profile (e.g., Ziegler et al., 2010). Accordingly, the finding that sensitivity to ARTs was related to all three domains of phonological processing suggested a fundamental relationship between acoustic processing of amplitude modulation/ARTs, phonological development, and literacy (see also Witton et al., 1998; Lorenzi et al., 2000). Since the initial investigation of ART (Goswami et al., 2002), many studies in different languages have investigated TS theory, using a range of different ART measures (see Supplementary Table S1 for a comprehensive summary). One difficulty with the original beat-based categorisation task used in 2002 was that it did not provide a value of rise time sensitivity for individual children, and further, it took a long time to administer (the stimuli were relatively long [~7 s] with a series of ARTs). Accordingly, other psychoacoustic tasks based on sine tones were developed to measure ART discrimination, for example, the ‘1 rise’ task (based on one sine tone with a single rise time) and the ‘2 rise’ task (based on a longer sine tone with two consecutive and identical rise times; Richardson et al., 2004). These tasks either utilised an AXB paradigm (1 rise, each stimulus ~800 ms, the rise time of either stimulus A or B differs from the standard stimulus X) or a 2IFC paradigm (two-interval forced choice, 2 rise, each stimulus ~3.5 s), respectively. Both the 1 Rise and 2 Rise tasks were subsequently employed in a range of studies across languages (summarised in Supplementary Table S1). Variability in the sensitivity of both tasks was found across different populations. The ‘2 rise’ task, in particular, showed an association with non-verbal IQ in some studies (e.g., Kuppen and Goswami, 2016) and produced null results regarding the expected impaired sensitivity in dyslexia in some languages (e.g., Greek, Papadopoulos et al., 2012). This may be because the nature of the ‘2 rise’ stimulus was more akin to a modulation detection task (see Wallaert et al., 2017) than an onset rise time task.

To mitigate some of these task-related issues, a 1-rise ART task based on speech-shaped noise (SSN) was developed by a group of researchers working in Dutch (e.g., Poelmans et al., 2011). The use of SSN is designed to address certain criticisms regarding using a sine tone stimulus to assess ART sensitivity. Temporal changes in a sound are accompanied by changes in its magnitude spectrum (Moore, 2003). An abrupt onset and offset of a sine tone will introduce frequencies known as ‘spectral splatter’. The sensitivity of the human auditory system in detecting such spectral changes provides a potential alternative cue to ART in the sine tone task. This can be ameliorated by reducing the rate of change of onsets and offsets in the sine tone task, as was indeed done in the sine tone studies (the 15 ms sine tone rise time used as a standard in the ‘1 rise’ task, e.g., Richardson et al., 2004). The wide spectrum of noise used for SSN removes the extraneous cue by masking the spectral splatter; accordingly, ART tasks based on SSN isolate the rise time parameter. The SSN ART task proved to have good sensitivity in studies of preschool children at family risk for dyslexia (Law et al., 2016; Vanvooren et al., 2017).

Other criticisms of the ‘1 rise’ sine tone ART task were that the duration of the steady-state portion of the stimulus changes as rise times get longer (shortening with longer rise times) and that the perceptual experience of intensity changes as rise times get shorter as sine tones with shorter rise times have more energy. Accordingly, sine tone ART stimuli provide alternative acoustic cues that could be used by the human auditory system as a basis for discrimination of ART differences. To remove these alternative cues, variations of the ‘1 rise’ task that either roved duration or intensity were developed, meaning that either duration or intensity varied constantly across stimuli, removing their utility as alternative acoustic cues (Huss et al., 2011; Goswami et al., 2013). In these investigations, group differences in performance for these roving tasks and the standard ‘1 rise’ task were similar for school-aged children, suggesting that these alternative acoustic cues were not being used as a cue in detection. Finally, an ART task based on synthetic syllables (/ba/ and /wa/) was developed in English to provide a more ecologically valid measure of sensitivity to ART in speech sounds (Goswami et al., 2011a). The ba/wa task (discussed further below) showed good sensitivity for children with dyslexia (Goswami et al., 2011a), leading to the development of the ‘ba rise’ task (Cumming et al., 2015). To the best of our knowledge, no study to date has compared the sensitivity of the different ‘1 rise’ AXB tasks (Sine Rise, SSN, ba Rise) in the same population of children. The current study closes that gap in the literature. Based on prior data, our first hypothesis (H1) was that ART thresholds would be elevated in children with dyslexia. We also expected that the a /ba/ rise task might be especially sensitive at younger ages, as it possesses the natural structure of voiced speech (hypothesis 2 (H2), see further below). A related hypothesis, H3, was that rise time discrimination by individual children would be similar regardless of the task format. This third hypothesis would predict significant relations between children’s thresholds in the three rise time tasks as different ART tasks should be measuring the same underlying sensory parameter of rise time sensitivity.

Regarding acoustic processing by children with dyslexia, ART processing is not the only sensory impairment. A meta-analysis of auditory non-speech studies conducted with both adults and children with developmental dyslexia found that group differences (participants with dyslexia worse than typically developing controls) were largest across studies for differences in frequency detection (weighted average effect size 0.7), duration detection (0.9), and ART discrimination (0.8; Hämäläinen et al., 2013). Uniquely, group deficits in ART discrimination were found in 100% of the studies in the meta-analysis, compared to 75% of the duration studies and 75% of the frequency studies. Longitudinal investigations of these other sensory parameters are largely absent from the literature across languages (see Witton et al., 2019, for a recent meta-analysis of frequency difference discrimination tasks in dyslexia). Intriguingly, Witton et al.’s meta-analysis identified a significant *positive* relationship between poorer (larger) frequency difference limen (measured as an effect size) and better non-word reading, the opposite of what would be expected if poorer sensitivity to frequency difference impairs phonological skills. Measuring additional sensory parameters longitudinally in children is important to gain a full picture of developmental relations between acoustic processing, phonological development, and literacy. In the current longitudinal report, we included psychoacoustic measures of sensitivity to rising frequency (which could also be considered spectrotemporal rise time), duration, and intensity, matching task format to the three ART tasks (we used a sine tone for the frequency rise task, an SSN stimulus for the intensity task, and a synthetic/ba/syllable for the duration task). Intensity discrimination has not been found to be atypical in previous studies of children with dyslexia and provides a useful control task for the attention and cognitive demands of psychoacoustic procedures (see Corriveau et al., 2010; Poelmans et al., 2011; Vanvooren et al., 2017; Flanagan et al., 2021). In earlier acoustic reports with the current cohort, we confirmed that the intensity difference limen for the CA and RA groups reduced with age (Flanagan et al., 2021), a phenomenon that has been found previously by others (e.g., Moore and Linthicum, 2007; Buss et al., 2009).

The decision to measure frequency rise perception by assessing sensitivity to a sine tone rising in frequency was made following a prior longitudinal study of acoustic processing by children with dyslexia reported by Goswami et al. (2013). Goswami et al. (2013) compared children’s sensitivity to rising versus falling frequency as related to phonology and musical beat perception using a non-speech tone task. In a study of 88 children, 38 children had dyslexia, and they reported that sensitivity to rising frequency was related to individual differences in both the phonological and musical tasks, whereas sensitivity to falling frequency was not. The children with dyslexia also showed significantly poorer sensitivity to rising frequency than age-matched control children. This finding differed from an earlier study with the same longitudinal cohort, which found that when speech sounds rather than sine tones were used to carry changes in ART versus rising frequency, children with dyslexia showed greater sensitivity to rising frequency than age-matched control children (Goswami et al., 2011a). Goswami et al. (2011a) compared ART discrimination when synthetic syllables either began with a plosive as in /ba/ or a glide as in /wa/. The change from /ba/ to /wa/ was created by either varying ART or by varying frequency rise time to investigate an alternative auditory theory of dyslexia based on discriminating rapid changes in frequency (Tallal, 2004). Goswami et al. (2011b) reported that children with dyslexia aged around 11 years were less sensitive to ART in making the ba/wa discrimination compared to age-matched control children but were significantly more sensitive to frequency rise time. This appeared to suggest that impaired auditory sensitivity to ART in dyslexia is accompanied by enhanced sensitivity to frequency rise time when synthesised speech is the input. A recent EEG study showed a similar pattern in infants at family risk (FR) or not at FR (NFR) for dyslexia, using modified synthetic speech ba/wa stimuli (Goetz et al., 2022). Mismatch response (MMR) data indicated that the FR infants could detect smaller frequency rise changes than the NFR infants, and the NFR infants could detect smaller ART changes than the FR infants. The /ba/ rise stimulus possesses the natural structure of voiced speech, unlike the sine tone and SSN ART stimuli, which have low ecological validity. As such, the acoustic structure of the /ba/ stimulus has a fundamentally different composition to the sine tone and SSN stimuli. In the current study, we measured sensitivity to rising frequency using a sine tone, following Goswami et al. (2013), to gain comparative data to the standard sine tone ART task. Based on Goswami et al. (2013), we expected (H4) that frequency rise thresholds would also be elevated in children with dyslexia.

To summarise, three ART tasks were employed in the current study, Ba Rise, SSN Rise, and Sine Rise tasks, along with three additional tasks assessing sensitivity to intensity, duration, and rising frequency. ART thresholds were expected to be elevated (worse) in children with dyslexia (H1). H2 was that the /ba/ rise task may be especially sensitive at younger ages, as it possesses the natural structure of voiced speech. H3 was that individual differences in ART sensitivity would be significantly related across the different task formats, as different ART tasks should be measuring the same underlying sensory parameter of rise time sensitivity, and H4 was that frequency rise thresholds should also be worse in children with dyslexia. Finally, individual differences in ART discrimination were expected to relate to individual differences in phonological processing and, consequently, literacy (H5). No other *a priori* predictions were made regarding relations between acoustic sensitivity and phonological development, given the mixed results reported in prior developmental studies.

The original research design planned to administer these six acoustic tasks at yearly intervals to a cohort of 127 children with and without dyslexia who had been recruited to participate in a 6-year longitudinal study of the rhythmic acoustic basis of dyslexia. Unfortunately, following recruitment and initial testing, the longitudinal research design was significantly impacted by the COVID-19 pandemic. Rather than the planned yearly assessments, during the pandemic and its aftermath, it was only possible to test the participating children with all six psychoacoustic tasks at three different measurement points over the course of the study (see Table 1) and with a reduced number of tasks at additional time points (see Table 1). Participating children also received a range of measures assessing language and phonological skills, literacy development, and non-verbal IQ, as detailed below. These measures enabled the investigation of both cross-sectional and longitudinal relations between acoustic processing of ART, frequency rise, duration, intensity, phonology, and literacy.

**Table 1:** Schedule of cognitive, language, and literacy measures taken at each testing time point.

Time point	Measures
Time Point 0 (Baseline) January–December 2018 *N* = 121 (CA = 30; DYS = 58; RA = 33)	BAS Reading & Spelling, TOWRE PDE & SWE, PhAB Rhyme, WISC Blocks
Time Point 1 January–July 2019 *N* = 121 (CA = 30; DYS = 58; RA = 33)	BPVS (receptive vocabulary) WISC Blocks Psychoacoustic thresholds for Rise time (3 tasks), Intensity, Duration, Frequency Rise.
Time Point 2 September–December 2019 *N* = 121 (CA = 30; DYS = 58; RA = 33).	BAS Reading & Spelling, TOWRE PDE & SWE PhAB Rhyme, Spoonerism, PhAB RAN (Picture, Digit), WISC Matrix, Digit Span Psychoacoustic thresholds for Rise time (3 tasks), Intensity, Duration, Frequency Rise.
Intervention 1 January–March 2020, followed by First UK lockdown	
Time Point 3 September–December 2020 *N* = 102 (CA = 30; DYS = 39; RA = 33)	BAS Reading & Spelling, TOWRE PDE & SWE PhAB Rhyme, Spoonerism, PhAB RAN (Picture, Digit) WISC Digit Span (Summer 2020) Psychoacoustic thresholds for Rise time (3 tasks), Intensity, Duration, Frequency Rise.
Second UK lockdown, followed by Intervention 2, January–March 2021	
Time Point 4 September–December 2021 *N* = 83 (CA = 30; DYS = 20; RA = 33)	BAS Reading & Spelling, TOWRE PDE & SWE, PhAB Rhyme, Spoonerisms, PhAB RAN (Picture, Digit) WISC Digit Span Psychoacoustic thresholds for Rise time (3 tasks), Intensity.
Intervention 3, January–March 2022	
Time Point 5 April–July 2022 *N* = 63 (CA = 30; RA = 33)	BAS Reading & Spelling, TOWRE PDE & SWE, BPVS PhAB Rhyme, Spoonerisms, PhAB RAN (Picture, Digit) WISC Digit Span Psychoacoustic thresholds for Rise time (3 tasks), Intensity, Duration.

BAS, British Ability Scales; TOWRE, Test of Word Reading Efficiency; PDE, phonemic decoding efficiency; SWE, Sight Word Efficiency; PhAB, Phonological Awareness Battery; WISC, Wechsler Intelligence Scale for Children; BPVS, British Picture Vocabulary Scales.

## 2 Methods

### 2.1 Participants

One-hundred and twenty-seven children were originally recruited for this study in 2018, although prior to the pandemic (which began closing UK schools in March 2020), six children (4 males and 2 female) had either dropped out or been excluded for low IQ (SS < 80). The remaining 121 children were tested on a yearly basis, with some of the children with dyslexia receiving oral rhythm-based interventions designed to improve phonological processing. The data reported here were collected in the first 5 school years of the study (2018–2022), with the first intervention delivered between test points 2 and 3 (see Table 1). Children with dyslexia were recruited via learning support teachers, who were informed that inclusion criteria for the children with dyslexia were participants to be free of any diagnosed learning difficulties aside from dyslexia (i.e., dyspraxia, ADHD, autistic spectrum disorder, speech, and language impairments). The language requirement was also assessed directly by administering a standardised vocabulary task, the British Picture Vocabulary Scales (BPVS, Dunn et al., 2009). The mean score for the children with dyslexia was 98 (see Table 2), which did not differ from the control children (102). Children were also required to have a non-verbal IQ of 80 or above and English as their first language. Children were assigned to the dyslexia (DYS) group if they scored at least 1 standard deviation below the test norm of 100 on (i) at least two of the 4 literacy measures, (ii) the phonology measure administered in the baseline screen (that was used to verify dyslexia status, described below), and (iii) had a non-verbal IQ of 80 or above. This approach was similar to previous studies (e.g., Kuppen et al., 2011) and was adopted as children without dyslexia in the UK typically score above the standardised mean of 100 on tests of literacy and phonology. There were 58 children who met the inclusion criteria (DYS group; 27 girls, mean age at recruitment 8 years, 1 month, UK school year 3, see Table 2). Control participants consisted of 30 chronological-aged-matched, typically reading children (CA; 10 girls, mean age at recruitment 7 years, 11 months, see Table 2) and 33 reading-level-matched children (RL; 18 girls, mean age at recruitment 6 years, 7 months, UK school year 1, see Table 2). Note, even though the DYS group was on average 2 months older than the age-matched controls, at Time Point 0, they were on average 19 months behind the CA group on the British Ability Scales (BAS) single word reading task (Elliott et al., 1996). All children had normal vision or corrected to normal vision with spectacles. All participants received a short hearing screen using an audiometer. Sounds were presented monaurally at a range of octave frequencies (0.5, 1.0, 2.0, and 4.0 kHz), and all participants were sensitive to sounds in the 20 dB HL range. In accordance with the Declaration of Helsinki, participant-informed assent and parental-informed written consent were obtained from all participants, and the study was approved by the Psychology Research Ethics Committee of the University of Cambridge.

**Table 2:** Participant characteristics by group for the behavioural tests of literacy, NVIQ, vocabulary, and phonology at baseline (Time Point 0), Time Point 1, and Time Point 2 (*N* = 121).

Mean (SD)	CA (*n* = 30)	DYS (*n* = 58)	RA (*n* = 33)	*F* (2,63.06)
Time Point 0 (Baseline)				
Age^1^	94.70 (4.23)	96.64 (5.33)	79.30 (9.55)	45.88***
Median (IQR)				H(2)
BAS Reading SS^2^	100.00 (7)	83.50 (8)	98.00 (6)	88.95***
BAS Spelling SS^2^	100.00 (7)	81.00 (5)	99.0 (7)	87.41***
TOWRE SWE SS^2^	109.00 (10)	80.50 (23)	107.50 (11)	69.13***
TOWRE PDE SS^2^	103.00 (10)	76.50 (13)	104.50 (12)	74.40***
PhAB Rhyme SS^2^	99.00 (16)	84.00 (14)	94.50 (14)	26.74***
WISC Blocks SS^3^	10.00 (3)	10.50 (4)	10.50 (3)	1.13
Time Point 1				
Mean (SD)				*F* (2, 68.67)
BPVS SS^4^	102.53 (11.75)	98.02 (12.50)	99.91 (8.43)	1.39
Time Point 2				
Median (IQR)	(*n* = 30)	(*n* = 58)	(*n* = 32)	H(2)
WISC Digit-Span SS^2^	9.00 (3)	7.00 (3)	10.00 (3)	24.94***
PhAB RAN Picture SS^5^	96.5 (15)	90 (23)	97.5 (22)	6.37*
PhAB RAN Digit SS ^2^	98.5 (16)	87.5 (22)	96.0 (21)	14.77***

^1^Welch F CA = DYS > RA. Bonferroni Adjusted for multiple tests.

^2^Kruskal–Wallis test. CA = RA > DYS. Bonferroni Adjusted for multiple tests.

^3^Kruskal–Wallis test. CA = RA = DYS. Bonferroni Adjusted for multiple tests.

^4^Welch F CA = RA = DYS. Bonferroni Adjusted for multiple tests.

^5^Kruskal–Wallis test. CA = DYS = RA. Bonferroni Adjusted for multiple tests. ***p < 0.001, **p < 0.01, *p < 0.05.

### 2.2 Procedure

Baseline screening was carried out in schools in 2018 (January–December) to identify participants with poorer reading skills and to recruit children for the two control groups (chronological-age-matched typically reading children, hereafter CA, and reading-age-matched typically reading children, hereafter RA). The baseline screening consisted of a phonological task (Phonological Assessment Battery [PhAB, Frederickson et al., 1997] Rhyme), four measures of reading and spelling (British Ability Scales [BAS, Elliott et al., 1996] Reading and Spelling scales; Test of Word Reading Efficiency [TOWRE, Torgesen et al., 1999], word [SWE, Sight Word Efficiency], and non-word [PDE, phonemic decoding efficiency] scales), and a non-verbal IQ measure (Wechsler Intelligence Scale for Children [WISC, Wechsler, 2016] Block Design). The baseline screen is subsequently referred to as Time Point 0 (see Table 1), and no auditory tasks were administered during the baseline screening session. Experimental assessments of auditory processing were subsequently administered at five time points over the project, as shown in Table 1 (January–July 2019, hereafter Time Point 1; September–December 2019, hereafter Time Point 2; September–December 2020, hereafter Time Point 3; September–December 2021, hereafter Time Point 4; and May–July 2022, hereafter Time Point 5). All assessments reported in the current manuscript were conducted in person when the schools were open in-between lockdowns (the UK lockdowns occurred in March–July 2020 and January–March 2021, in addition many of our participating schools did not allow researchers back into classrooms until September 2021). A schematic overview of which tasks were administered in each assessment is provided in Table 1. Note that after the pandemic, it was more difficult to get into schools due to their COVID safety procedures. Accordingly fewer tasks could be delivered during a school term. Thereafter, we focused largely on the ART measures as these were our core theoretical interest and we were testing a series of *a priori* predictions. We had to drop the frequency rise task at Time Points 4 and 5, as well as the duration task at Time Point 4. Furthermore, the 58 children with dyslexia were randomly assigned to three intervention groups when the project began (19, 19, and 20 per group, respectively); hence, some of the children with dyslexia received a rhythm-based oral intervention in the school Spring Terms (January–March) of either 2020, 2021, or 2022. As it was not the focus of this study, once a child had received an intervention, their psychoacoustic and behavioural data were omitted from the group comparisons and longitudinal analyses reported in this manuscript. This was because an effective intervention would also be expected to impact acoustic and phonological development. Therefore, the participant numbers at each time point were as follows: Time Point 1, *N* = 121 (CA = 30; DYS = 58; RA = 33); Time Point 2, *N* = 121 (CA = 30; DYS = 58; RA = 33); Time Point 3, *N* = 102 (CA = 30; DYS = 39; RA = 33); Time Point 4, *N* = 83 (CA = 30; DYS = 20; RA = 33); Time Point 5, *N* = 63 (CA = 30; RA = 33).

### 2.3 Phonological processing

Phonological processing was assessed using three measures from the standardised Phonological Assessment Battery (PhAB; Frederickson et al., 1997), Rhyme, Spoonerisms, and Rapid Automatised Naming (RAN). In the PhAB Rhyme task, children are asked to identify the rhyme in single-syllable real words. Three practice trials are given with feedback. The experimenter reads out three words to the child. The task is to select the two words that rhyme, i.e., sound the same at the end (e.g., *sail*, boot*, nail;* big*, hiss, miss*). The test was scored out of 21 trials, and the child’s score out of 21 was used for subsequent analyses. The PhAB Spoonerisms test is timed and comes in two parts, each lasting a maximum of 3 min. The first part consists of 10 semi-spoonerisms, where the first phoneme of a word is replaced by a new one, e.g., ‘dog’ with a /l/ gives? – ‘log’. The second part consists of 10 full spoonerisms, where the task is to exchange the leading sounds of two words, e.g., ‘lazy dog’ gives… (answer: ‘daisy log’). A point is given for each correct word. The raw score is out of 30, and the child’s raw score was used for subsequent analyses.

PhAB RAN consists of two parts: a picture naming speed task and a digit naming speed task. In the picture naming task, line drawings of five common objects are to be named as quickly and accurately as possible. To begin, the child is familiarised with the five drawings of objects (ball, hat, door, table, and box). Then, they are asked to name them from a sheet with 50 of the objects (5 rows x 10 columns) in a randomised order. This is repeated with a second set of 50. The raw score is the sum of the two times in seconds. For digit naming, the eight single-syllable digits (1,2,3,4,5,6,8,9) are used. After familiarisation with these digits, a list of 50 digits (10 x five-digit numbers) is to be read out as quickly as possible. This is repeated with a second set of 50. The raw score is the sum of the two times, in seconds, and was used for subsequent analyses.

Phonological short-term memory (PSTM) was assessed using the Wechsler Intelligence Scale for Children fifth edition (WISC-V) digit span test. In this test, children listen to a sequence of numbers read aloud by the researcher. The test consists of three types where the child must repeat back the sequence in one of three ways: in the same order, the reverse order, or ascending order. Each test item consists of two trials. The first trial consists of two numbers (e.g., 2–9) and is increased by one number on subsequent items. Each test type is discontinued when the child incorrectly answers both trials in an item. The tests start with a practise for the reverse and ascending sequences. One point is given for each correctly responded item. The maximum raw score for digit span is 54, and standard scores are normalised to have a mean of 10 with a standard deviation of 3.

### 2.4 Standardised tests of language

#### 2.4.1 Vocabulary

The British Picture Vocabulary Scale (BPVS; Dunn et al., 2009) is a test of receptive vocabulary. In the test, the child is shown 4 pictures on a page in a stimulus book. The child must point to the picture that best illustrates the meaning of the word spoken by the experimenter. The maximum raw score is 168, and a standard score is computed (mean of 100, S.D. 15).

#### 2.4.2 Standardised tests of literacy

Reading and spelling ability was measured using the BAS single-word reading and spelling subscales (Elliott et al., 1996). Sight word reading efficiency (SWE) and phonemic decoding efficiency (PDE) were measured using the Test of Word Reading Efficiency (TOWRE) (Torgesen et al., 1999). The TOWRE is a timed measure of reading accuracy, which has two parts. Each part is limited to 45 s, in which the child reads aloud as many words (Sight Word Efficiency, SWE) or non-words (Phonemic Decoding Efficiency, PDE) as quickly and as accurately as possible. For the correlational and longitudinal analyses reported here, raw scores were used, as they were more sensitive to small differences among children (see Kyle et al., 2013) and as standard scores for all participants fell during the COVID-19 pandemic.

### 2.5 Standardised cognitive ability tests

Children were given the Block Design subscale of the Wechsler Intelligence Scales for Children – Fifth UK Edition (WISC-V; Wechsler, 2016). In the *Block Design* task, the child must recreate, within a specified time limit, the design of a hand-made model or picture in a stimulus book using red and white blocks. There are 14 items with a total maximum score of 68. A scaled score between 1 and 19 can then be computed. The mean scaled score is 10.

### 2.6 Experimental psychoacoustic tasks

The psychoacoustic tasks were programmed by the first author using Neurobehavioral Systems Presentation® software^1^ and run on a laptop computer (Lenovo T480). Sounds were produced via an AudioQuest DragonFly® soundcard, presented binaurally through Sennheiser HDA 300 headphones. A calibration verification program was run at the start of each school session. The acoustic stimuli were presented at 75 dB sound pressure level (SPL) unless roved in level. Rise tasks with different spectral content were equated for loudness using a loudness model for time-varying sounds (Moore et al., 2016). Children’s responses were made using the left and right buttons on an X-Box® games controller connected via USB to the laptop.

Psychoacoustic tasks using non-speech tokens (sine tone or speech-shaped noise) or speech stimuli (the syllable/ba/) were used to measure sensitivity to sound intensity, duration, frequency rise, and ART. The auditory tasks developed for this study were all administered as child-friendly computer games based on the AXB paradigm “Dinosaur” threshold estimation program originally developed by Dorothy Bishop (Oxford University). Three sounds were played sequentially, with 500 ms inter-stimulus intervals (ISIs). The second sound (X) was always the reference or standard sound, and either the first (A) or the third (B) was the target stimulus. A practice session of five trials with the easiest condition was run at the start of each type of stimulus, and the difference to listen for (e.g., intensity or sound onset) was explained to the child. Feedback was given via onscreen picture tokens for each correct response. The threshold estimation program used an adaptive staircase procedure (Levitt, 1971) with an initial 2-down 1-up procedure followed by a 3-down 1-up procedure after two reversals. The initial step size was eight, which halved after the fourth and sixth reversals. A run of the program terminated after the eighth reversal or 40 trials, whichever occurred first. The threshold score was calculated using the mean of the last 4 reversals.

#### 2.6.1 Amplitude rise time

To address the main research question, three measures of ART processing were presented using different temporal fine structures, a sine tone task, a speech-shaped noise task, and a /ba/ speech token task.

##### 2.6.1.1 Sine rise task

The sine ART task used the same stimuli from previous “1 Rise” studies (e.g., Goswami et al., 2013). The stimuli were three 500-Hz tones of 800 ms duration separated by two 500-ms ISIs. A standard tone, which was presented twice, had a 15-ms linear onset rise time, a 735-ms steady state portion, and a 50-ms linear fall time. The target tone had a variable onset rise time, with the initial and longest rise time being 300 ms. The target stimuli rise time ranged in 39 linear steps of 7.3 ms from 300 ms to 15 ms. The computer screen displayed three cartoon dinosaurs in a row in an AXB format. The target tone would appear in either the first or third interval, with the other two being the standard tone. The experimenter explained to the participant that the cartoon dinosaurs would each jump and make their sounds in turn. The target could be either A or B. X was always the standard, along with one of B or A. Participants were to decide which of A or B dinosaur’s sound was different from the other two, having a slow or softer beginning. Feedback from on-screen tokens was given to indicate correct responses. The experimenters’ verbal instructions were reinforced with 5 practice trials with feedback, with the target rise time at the maximum value of 300 ms.

##### 2.6.1.2 Speech shaped noise (SSN) task

In this study, unmodulated speech-shaped noise (SSN) with the long-term average spectrum of speech (CCITT Rec. 227) was created from Gaussian noise using MATLAB ccitt_filter function (Seeber, 2005). The inherent modulations found in the envelope of Gaussian noise were controlled by selecting unique samples from the noise for each target token. To avoid learning the timbre of the noise, there were 4 unique samples for the standard tokens. The envelope of the SSN tokens was identical to that of the standard and target tones used in the Sine Rise task, i.e., the overall duration was 800 ms, linear offset ramps of 50 ms, linear onset rise time for the standard of 15 ms, and 15–300 ms for the target. The SSN onset rise time varied in 39 logarithmically spaced steps. The wideband noise difference limen is in the range of 0.5–1 dB (Moore, 2003). To remove small loudness differences between tokens as a potential cue, the level of the target stimuli was pseudo-randomly varied between 75 and 73 dB. The levels of the standard stimuli were also pseudo-randomly roved between 75 and 72 dB. In total, there were 16 standard stimuli (4 levels x 4 noise samples).

##### 2.6.1.3 Ba rise task

The /ba/ stimuli were based on those used in the study by Cumming et al. (2015), with the addition of random level roving of the standard stimuli in the range of ±2.25 dB to remove the loudness level as an alternative cue to identification. The stimuli were based on the /ba/ syllable, which was 300 ms long with a flat f_0_ (fundamental frequency) of 200 Hz. The standard stimulus (X) had a 10-ms amplitude onset rise time. The target stimulus had an onset rise time that ranged from the longest rise time at 150 ms to the shortest of 10 ms, in steps of 3.7 ms.

#### 2.6.2 Other psychoacoustic measures

There were also 3 psychoacoustic tasks designed to measure sensitivity to intensity, duration, and rising frequency, which were matched for task format to the three ART tasks as follows:

##### 2.6.2.1 Intensity task

The stimuli consisted of unmodulated speech-shaped noise (SSN) with the long-term average spectrum of speech (CCITT Rec. 227). This was created from Gaussian noise using MATLAB (see Flanagan et al., 2021, for stimulus generation). Each stimulus had a duration of 800 ms, with 50 ms linear amplitude rise time and 50 ms offsets. The intensity of the two reference tones was 64 dB SPL. The intensity of the target interval ranged from 74 to 64 dB SPL in 40 steps of 0.25 dB SPL. Participants were introduced to three cartoon dinosaurs. It was explained that each would make a sound in turn, and the task was to decide which dinosaur was the loudest. The threshold estimation program used an adaptive staircase procedure (Levitt, 1971) with an initial 2-down 1-up procedure followed by a 3-down 1-up procedure after two reversals. The initial step size was eight, which halved after the fourth and sixth reversals. A run of the program terminated after the eighth reversal or 40 trials, whichever occurred first. The threshold score was calculated using the mean of the last 4 reversals.

##### 2.6.2.2 Duration task

This task was derived from the adaptive threshold estimation AXB/ba/ speech duration task by Cumming et al. (2015). In this study, the /ba/ tokens were also roved in level in the range of ±2.0 dB to rule out loudness as a potential cue. The /ba/ stimuli had a flat f0 of 200 Hz. The standard stimulus (X) had a duration of 150 ms. The variable target stimulus (either A or B) was from a range of 39 durations with a maximum duration of 300 ms in 3.9 ms steps. The task was to identify which cartoon sheep made the /ba/ longer than the other two.

##### 2.6.2.3 Frequency rise task

This task was based on the rising frequency of AXB tasks by Cumming et al. (2015). The three stimuli were sinusoidal tones of 300 ms duration, with linear onset and offset ramps of 5 ms. The standard stimulus (X) consisted of a sine tone that started at 295 Hz and increased in frequency to 500 Hz over a period of 10 ms (i.e., frequency rise time). The target stimulus (either A or B) had a frequency rise time that ranged over 39 durations as an exponential function from the shortest rise time (10 ms) to the longest rise time (150 ms). Starting with the longest target rise time, three cartoon elephants were displayed on the screen. Each moved in turn with the three sound stimuli. The child was asked to decide which elephant ‘trumpeted’ more slowly than the other two. The standard stimuli were roved over ±0.75 dB to eliminate possible loudness cues due to differences in frequency content. As noted earlier, this task could be considered a measure of sensitivity to spectrotemporal rise time.

### 2.7 Analysis strategy

Study data were collected and managed using REDCap electronic data capture tools hosted at the University of Cambridge (Harris et al., 2019). Data exploration was conducted using SPSS (ver. 28.0). Data Analysis was conducted using SPSS (ver. 28.0), R (R Core Team, 2020), and RStudio (RStudio Team, 2020).

#### 2.7.1 Cross-sectional analysis

A series of cross-sectional analyses at Time Points 0 to Time Point 4 were conducted to investigate group characteristics for the dyslexic and control children. The influence of age on the performance in the acoustic tasks was investigated using a series of simple linear regressions. As age exerted significant effects, longitudinal regression analyses related to the development of phonology and literacy were conducted with the dyslexic and CA-matched control children only.

#### 2.7.2 Longitudinal analysis strategy

To investigate the main effects and the interaction of Time Point (TP) and group membership (CA, DYS, RA) on the development of the auditory threshold measures, linear mixed effect models were run for each of the outcome variables Sine Rise, SSN Rise, Ba Rise, Frequency Rise, Intensity, and Duration, using the packages lme4 (Bates et al., 2015) and lmerTest (Kuznetsova et al., 2017) in R (R Core Team, 2020). This enabled a systematic comparison of our groupings (CA, DYS; CA, RA; DYS, RA) and their performance at the different TPs 1–3.

The formula to test for main effects and interaction was in the form: model = (outcome variable ~ Time Point*Group + (1| Participant), data). Significance levels were determined using the Type III Analysis of Variance with Satterthwaite’s method.

As might be expected, the different measures of phonological awareness and literacy were highly inter-correlated. Accordingly, a series of composite variables based on raw scores was created using the Percent of Maximum Possible transformation (Cohen et al., 1999). This transform is suitable for longitudinal studies as it maintains the longitudinal relationships in the data. To create the Literacy composite variable, scores on the BAS Reading, BAS Spelling, TOWRE PDE, and TOWRE SWE were combined for each time point (LIT_TP0 – LIT_TP5). The same approach was used to create the composite Phonological Awareness measure (combining PhAB Rhyme and Spoonerism raw scores; PA_TP2 – PA_TP5). The PhAB RAN Picture and Digit subscales used the same scale, so raw scores were simply averaged to produce the composite scores RAN_TP2–RAN_TP5. Finally, the WISC-V digit span raw score was used to measure PSTM.

## 3 Results

We first report descriptive data regarding participant characteristics and age effects (see sections 3.1–3.4) and then investigate our hypotheses H1–H5 (sections 3.5 and 3.6).

### 3.1 Participant characteristics

Group data regarding the behavioural measures are presented in Table 2. The data from these behavioural tasks, the ART tasks, and the frequency rise, intensity, and duration tasks were explored for assumptions of normality. A Shapiro–Wilk test revealed that the data from the psychoacoustic tasks, WISC Blocks, BAS Spelling, TOWRE PDE, and PhAB Rhyme at Time Point 0 were not normally distributed. Hence, the independent samples Kruskal–Wallis test (non-parametric one-way ANOVA) was used to test for group differences in the behavioural measures. Bonferroni correction was applied for multiple comparisons.

Inspection of the group comparisons presented in Table 2 shows that at the time of the baseline screen (Time Point 0), the children with dyslexia were significantly impaired in the literacy and phonological awareness measures (PhAB rhyme) compared to both the CA and RA control children. The further experimental measures taken at Time Points 1 and 2 (receptive vocabulary [BPVS], RAN, WISC vocabulary, and WISC digit span) confirm that the DYS group had impairments in phonological processing, PSTM, and RAN but not in vocabulary development. Supplementary Table S2 is provided to show chronological age and reading ability by group for Time Points 2, 3, and 4 for the entire baseline cohort that were available at each subsequent time point for testing. The ability ages demonstrate that the children with dyslexia fell further behind their typically developing peers in the literacy measures as the study progressed.

### 3.2 Effect of age on threshold levels

To investigate the effect of age on auditory thresholds, a cross-sectional analysis was performed. For the 63 typically developing children (CA and RA), a series of six simple linear regressions were conducted. Age at Time Point 1 was the predictor for amplitude rise time thresholds (Sine and Ba) and also for the thresholds for frequency rise, duration, and intensity. Age at Time Point 2 was the predictor for SSN Rise at Time Point 2. The results of these analyses are presented in Supplementary Table S3. All auditory thresholds were significantly predicted by age. In the case of SSN at Time Point 1, there were floor effects, which may be due to the added complexity of employing roving stimuli and noise standards. This appeared to make the task more difficult for all participants, at least initially, which could account for the lack of correlation with age.

### 3.3 Auditory threshold changes over Time Points 1 to 5

**Figure 1:**
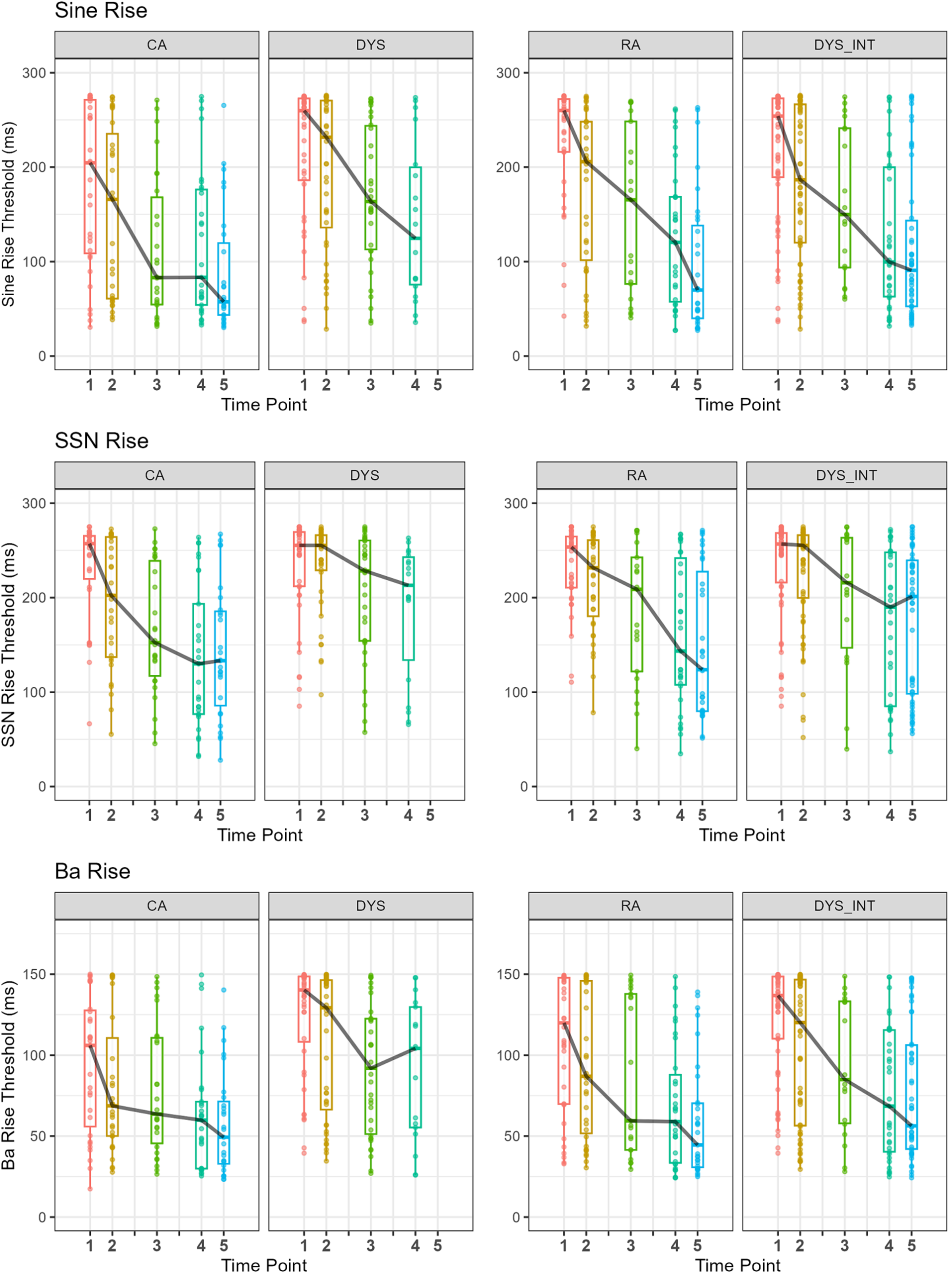
Boxplots and individual rise time thresholds for all participants by group (CA, DYS, and RA) at each time point. For comparison purposes, DYS TP1 to TP3 show the subset of DYS children included in the longitudinal analysis who had not received an intervention (i.e., subgroups 2 and 3). DYS TP4 shows subgroup 3. There are no DYS children at TP5, as all had received an intervention. DYS_INT at TP1 and TP2 show all DYS children (i.e., intervention subgroups 1, 2, and 3), and for time points after, those who had taken part in an intervention (TP3 subgroup 1, TP4 subgroups 1 and 2, and TP5 subgroup 1, 2, and 3). Black line links the boxplot median value. Note the different scales for Ba Rise.

**Figure 2:**
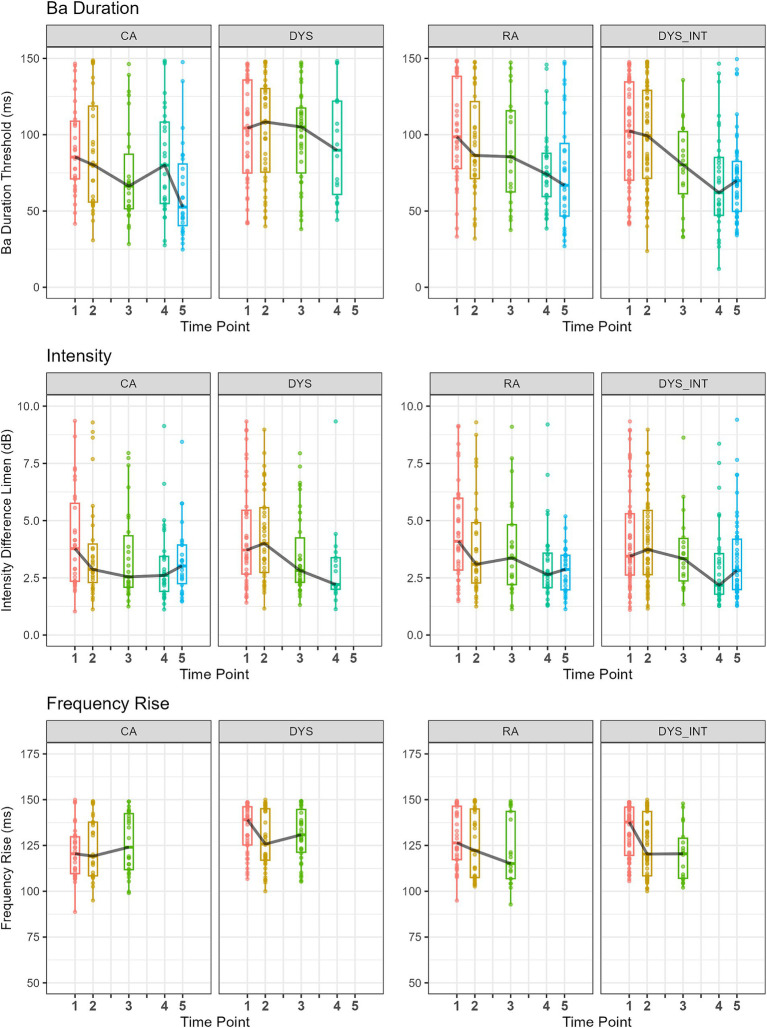
Boxplots and individual thresholds for Duration, Intensity Difference Limen (DL), and Frequency Rise for all participants by group (CA, DYS, and RA) at each time point. DYS TP1 to TP3 show the subset of DYS children included in the longitudinal analysis who had not received an intervention (i.e., subgroups 2 and 3). DYS TP4 shows subgroup 3. There are no DYS children at TP5, as all had received an intervention. DYS_INT TP1 and TP2 show all DYS children (i.e., intervention subgroups 1, 2, and 3), and for time points after those who had taken part in an intervention (TP3 subgroup 1, TP4 subgroups 1 and 2, and TP5 subgroup 1, 2, and 3). Black line links the boxplot median value.

To capture developmental changes in auditory processing at the level of individual children, Figure 1 depicts the ART threshold data for Time Points 1, 2, 3, 4, and 5. As some DYS children received an intervention following Time Point 2, two sets of plots are included for children with dyslexia (DYS, DYS_INT). Two columns are included for the children with dyslexia to account for possible differences in sensitivity following the oral rhythmic training and to show the DYS children who were included in the longitudinal analysis. The figure shows boxplots and individual threshold data for each participant by group (CA, DYS, RA, DYS_INT). Column DYS at Time Points 1 to 3 shows the two subgroups of children with dyslexia who had received no intervention by Time Point 3, followed by the final no-intervention subgroup at Time Point 4. Column DYS_INT shows all DYS children up to and including Time Point 2, followed by time points for the DYS children who received an oral rhythmic intervention, which theoretically could also help their ART processing. The ART tasks are shown by row (Sine, SSN, and Ba). Occasionally, performance in the adaptive tasks was too poor for a threshold to be computed. In these cases, to avoid missing data, a maximum threshold value was applied. For the Sine ART task, this procedure was followed for 5 participants (3 DYS, 2 RA) at Time Point 1 and 1 participant (DYS) at Time Point 2. For the SSN task, it was followed for 9 participants at Time Point 1 (2 CA, 3 DYS, and 4 RA) and 2 participants (1 DYS and 1 RA) at Time Point 3. For the Ba Rise task, the threshold procedure was followed for three children at Time Point 1 and three children at Time Point 2 (2 DYS and 1 CA). For comparison purposes, Figure 2 provides auditory threshold data for the other three tasks: frequency rise, duration, and intensity. Again, when a threshold could not be computed, a maximum threshold value was applied. This only occurred for the duration of the task for one child at Time Point 1 (1 RA).

### 3.4 Longitudinal effects of Time Point and group on auditory thresholds

For the longitudinal analyses (CA and DYS only), a linear mixed effect model was run, including Time Points 1, 2, and 3. The CA group was comprised of *n* = 30, and to meet the requirements of the LME regression model, the dyslexic group included only those children who would not have received an intervention by Time point 3 (*n* = 39). For Sine Rise thresholds, there was a significant main effect of Time Point (TP1–TP3) [*F*_(2,124.88)_ = 15.22 *p* < 0.001] with thresholds decreasing with time point. There was a significant main effect of group (CA, DYS) [*F*_(1,68.45)_ = 8.53, *p* < 0.01] with lower thresholds for CA than DYS, and there was no significant interaction of Time Point by Group (*p* = 0.65). For the SSN Rise thresholds, there was a significant main effect of Time Point (TP1–TP3) [*F*_(2,127.58)_ = 13.52 *p* < 0.001] with thresholds decreasing with time point, and a significant main effect of group (CA, DYS) [*F*_(1,68.21)_ = 5.88, *p* = 0.017] with lower thresholds for CA than DYS. There was a significant interaction of Time Point by Group [*F*_(2,127.58)_ = 3.32, *p* = 0.039], reflecting the greater rate of threshold decrease for the CA group. For Ba Rise thresholds, there was a significant main effect of Time Point (TP1 – TP3) [*F*_(2,125.89)_ = 13.19 *p* < 0.001] with thresholds decreasing with time point and a significant main effect of Group (CA, DYS) [*F*_(1,67.45)_ = 9.04, *p* < 0.01], reflecting lower thresholds for CA than DYS. There was no significant interaction of Time Point by Group (*p* = 0.30). For Frequency Rise, there was no significant effect of Time Point (*p* = 0.69), but there was a significant main effect of Group (CA, DYS) [*F*_(1,66.23)_ = 5.37, *p* = 0.024], reflecting the lower thresholds seen for the CA group. There was a significant interaction of Time Point by Group [*F*_(2,124.64)_ = 3.40, *p* = 0.021], reflecting the reduction in thresholds with time for the DYS group only. For Duration, there was a marginal effect of Time Point (*p* = 0.054) with a trend towards lower thresholds, and there was a significant main effect of Group (CA, DYS) [*F*_(1,68.13)_ = 7.23, *p* < 0.01] with thresholds for CA lower than for DYS. There was no significant interaction of Time Point by Group (*p* = 0.22). For Intensity, there was a significant main effect of Time Point [*F*_(2,125.49)_ = 4.34, *p* = 0.015], reflecting the reduction in thresholds with time. There was no significant effect of Group (CA, DYS) (*p* = 0.39) and no significant interaction of Time Point by Group (*p* = 0.78).

### 3.5 Differential task effects

As will be recalled, *a priori*, we predicted that ART thresholds would be elevated in children with dyslexia (H1), that the Ba Rise task may be especially sensitive at younger ages as it possesses the natural structure of voiced speech (H2), and that ART discrimination by individual children would be similar whatever the task format (H3). Inspection of Figure 1 (ART) and the results of the linear mixed effects model for Time Points 1 to 3 above shows that the typically developing children in the CA group achieved lower (better) thresholds than children of the same age in the DYS groups, over time and as a group, for all the ART measures. This is supportive of H1. The younger RA group was not significantly different from children in the DYS group for the Sine ART measure and had marginally higher thresholds for the SSN (*p* = 0.09) and Ba Rise (*p* = 0.09) ART measures. A linear mixed effects model comparing rise time thresholds for the CA group with the younger RA group showed similar sensitivity to the older typically developing children for the Ba Rise task at Time Points 2 and 3 (*p* = 0.19). This suggests that the Ba Rise measure is most sensitive at younger ages, consistent with H2. By Time Point 4, at which point, the younger RA group was now also 18 months ahead of the DYS group in reading age (see Supplementary Table S2), their Sine Rise sensitivity was not significantly different to the older DYS children (Mann–Whitney U = 218.00, *p* = 0.44). This may suggest that normative development of ART sensitivity is not dependent on learning to read. Nevertheless, a range of performance is visible in all groups across all time points.

Regarding acoustic sensitivity in the other psychoacoustic tasks, the analysis above and Figure 2 demonstrate clearly that all three groups showed similar intensity thresholds at all time points. This suggests that the cognitive demands of performing psychoacoustic tasks cannot explain the group differences in ART sensitivity. The groups also showed no significant effect of Time Point for the Frequency Rise task across the first three time points, with the CA having significantly lower median thresholds than the DYS. The DYS group also showed the least developmental progress in the Duration measure. Inspection of Figure 2 suggests that the children with dyslexia who were receiving the oral rhythm-based intervention improved in their sensitivity to duration as the study progressed. However, this is beyond the scope of the current analysis. Further visualisation of these developmental differences is provided in the Supplementary Figures S1, S2.

Group thresholds for the psychoacoustic tasks at Time Points 1 and 3 are presented in Table 3. Mann–Whitney 1-way ANOVAs comparing the DYS and CA groups showed that there was no significant difference between the groups regarding intensity discrimination at either test point, but there were some group differences for the other acoustic measures. At Time Point 1, the CA and DYS groups differed significantly on Ba Rise and Frequency Rise measures, supporting H1, H2, and H4, that frequency rise thresholds would also be elevated in children with dyslexia. By Time Point 3, the CA and DYS groups differed significantly on SSN Rise and Sine Rise, supporting H1, but no longer differed in their sensitivity to Ba Rise and Frequency Rise. The children with dyslexia were less sensitive to differences in duration than the CA group by Time Point 3.

**Table 3:** Mann–Whitney non-parametric ANOVAs comparing CA with DYS psychoacoustic thresholds (Median, IQR) for groups at Time Point 1 and Time Point 3.

Time Point 1	CA (*n* = 30)	DYS (*n* = 58)	Mann–Whitney U
Sine Rise (ms)	205.37 (167.11)	250.70 (86.71)	1031.0
SSN Rise (ms)	257.69 (55.09)	255.42 (56.47)	916.5
Ba Rise (ms)	105.91 (77.97)	136.29 (40.50)	1244.0***
Frequency Rise (ms)	119.37 (21.52)	138.8 (27.72)	1217.5**
Intensity (dB)	3.83 (3.63)	3.45 (2.67)	871.0
Duration (ms)	85.28 (38.67)	100.94 (62.07)	984.0
Time Point 3	CA (*n* = 25)	DYS (*n* = 33)	
Sine Rise (ms)	83.60 (136.49)	163.58 (131.68)	579.0^**^
SSN Rise (ms)	150.26 (131.42)	229.23 (107.19)	561.0^*^
Ba Rise (ms)	61.06 (68.93)	88.19 (72.90)	485.0
Frequency rise (ms)	129.05 (32.44)	129.42 (24.49)	466.5
Intensity (dB)	2.53 (2.42)	2.83 (2.45)	463.0
Duration (ms)	64.90 (31.70)	107.21 (50.73)	603.0^**^

*p < 0.05, **p < 0.01, ***p < 0.001 (two-tailed).

#### 3.5.1 Art sensitivity as measured by different tasks

To investigate the hypothesis that the different ART tasks should be measuring the same underlying sensory parameter of rise time sensitivity (H3), we next explored the relations between the different acoustic measures of ART using directional (one-tailed) Spearman correlations. Correlations between the different ART measures are shown in Table 4 for the age-matched children only (CA and DYS). As can be seen by inspecting Table 4, all the different ART measures were significantly positively correlated with each other, with the exception of Ba Rise with Sine Rise at Time Point 4. However, by Time Point 4, only 17 children with dyslexia were contributing data to these correlations.

**Table 4:** Spearman correlations between the ART and frequency rise measures administered at Time Points 1, 2, 3, and 4, controlling for age and NVIQ.

	Sine Rise	SSN Rise	Ba Rise
Time Point 1 (*N* = 88)			
1. Sine Rise	-		
2. SSN	0.357***	-	
3. Ba Rise	0.371***	0.374***	-
4. Freq Rise	0.346***	0.324***	0.417***
Time Point 2 (*N* = 88)			
1. Sine Rise	-		
2. SSN	0.448***	-	
3. Ba Rise	0.361***	0.291**	-
4. Freq Rise	0.509***	0.253*	0.405***
Time Point 3 (*N* = 58)			
1. Sine Rise	-		
2. SSN	0.526***	-	
3. Ba Rise	0.350**	0.444***	-
4. Freq Rise	0.470***	0.327**	0.414**
Time Point 4 (*N* = 50)			
1. Sine Rise	-		
2. SSN	0.363**	-	-
3. Ba Rise	0.072	0.397**	-

CA and DYS only.

*Correlation significant at 0.05 level (1-tailed). **Correlation significant at 0.01 level (1-tailed). ***Correlation significant at 0.001 level (1-tailed).

**Figure 3:**
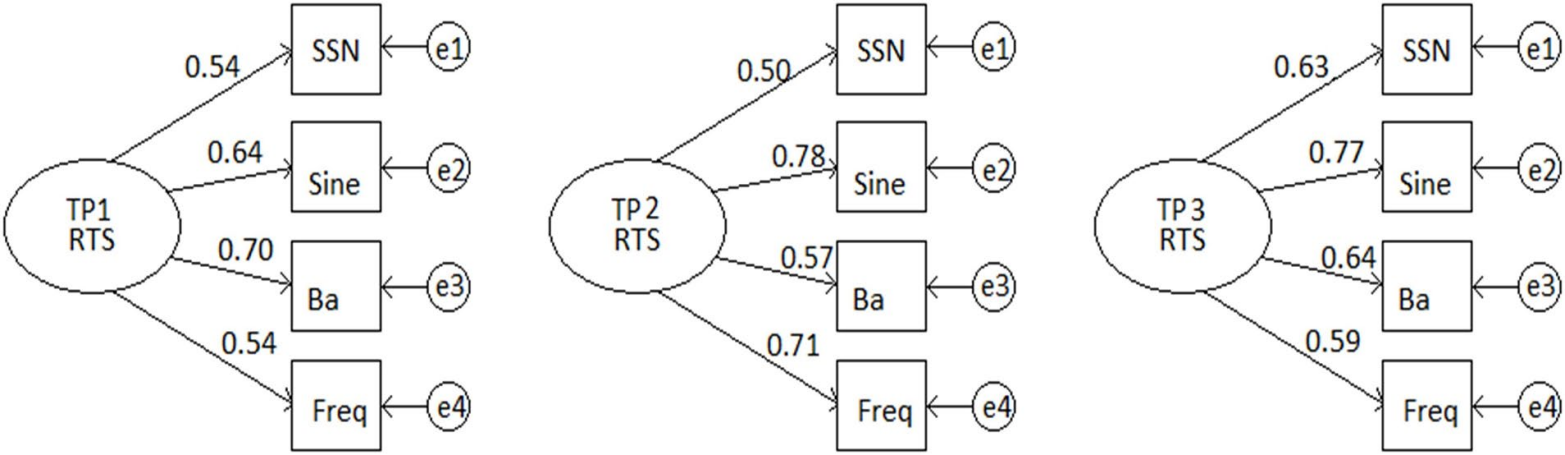
Single factor models of latent variable rise time sensitivity (RTS) and the observed indicators SSN Rise, Sine Rise, Ba Rise, and Frequency Rise at Time Points 1, 2, and 3 for the age-matched participants CA and DYS only.

Rise time sensitivity could also be considered a latent variable. A measure of internal consistency reliability based on correlations, Cronbach’s coefficient alpha, was thus calculated in SPSS for the different rise time measures Sine Rise, SSN Rise, Ba Rise, and Frequency Rise. Frequency Rise was included in the analyses as it assesses sensitivity to spectrotemporal rise time. The reliability of these scores, alpha, was 0.69 at TP1, 0.73 at TP2, and 0.75 at TP3. Scores of 0.7 or above are considered acceptable for the exploratory nature of our research question (Nunnally, 1978). However, a criticism of Cronbach’s alpha is that it is sensitive to assumptions of unidimensionality and tau-equivalence (Hayes and Coutts, 2020). Hence, a more robust method of measurement reliability, McDonald’s omega (ω), which does not rely on these assumptions, was also estimated. A single factor model for the latent variable Rise Time Sensitivity (RTS) for variables Sine Rise, SSN Rise, Ba Rise, and Frequency Rise was fitted using SPSS using Maximum Likelihood estimation for the first three time points (TP1–TP3) where all four rise measures were taken. The goodness of fit test showed the data were not significantly different from the model for each time point (TP1, χ^2^(2) = 3.09, *p* = 0.86; TP2, χ^2^(2) = 2.34, *p* = 0.31; TP3, χ^2^(2) = 3.21, *p* = 0.20). The factor loadings λ_i_ and the error variances *V(e_i_)* can be found in Supplementary Table S5. The unidimensional factor models of latent variable rise time sensitivity (RTS) and the observed indicators are shown in the measurement models in Figure 3.

The standardised factor loadings for each of the variables were used to explore the factorial structure of the data by calculating McDonald’s omega (ω), following the approach of Hayes and Coutts (2020) as follows:


(1) $$\omega =\frac{{\left(\sum \lambda_{i}\right)}^{2}}{\left({\left(\sum \lambda_{i}\right)}^{2}+\sum V\left(e_{i}\right)\right)}$$

Where λ_i_ are the standardised factor loadings and *V(e_i_)* is the error variance for the *i^th^* item.

McDonald’s omega was calculated using Equation (1) above, and values were as follows: TP1 ω = 0.70; TP2 ω = 0.74; and TP3, ω=.75. Note that the values of omega are very close to the corresponding Cronbach’s alpha, supporting the view that the observed measures were measuring the same (unidimensional) latent variable and were tau-equivalent.

### 3.6 Auditory sensitivity and phonological development

We next focussed on H5, which shows that ART sensitivity would show significant relations to phonological development. We also explored potential developmental relations for the psychoacoustic measures of sensitivity to intensity, duration, and frequency rise, for which no *a priori* predictions were made. To minimise the number of variables, we created a series of composite variables as described above for phonological awareness, RAN, and literacy and also included PSTM as an independent phonological measure. First, cross-sectional correlations at Time Point 2 between acoustic sensitivity and the composite measures were computed and are shown in Table 5 (controlling for age and NV IQ; CA and DYS only). Inspection of the table reveals that the Sine Rise, SSN Rise, and Ba Rise ART measures all showed significant concurrent relations with phonological awareness and with literacy based on our directional (one-tailed) hypothesis. Sine Rise also showed a significant (one-tailed) concurrent association with RAN. Lower auditory threshold values were indicative of smaller, just noticeable differences and were associated with better performance. Regarding the other acoustic tasks, there were no concurrent relations with phonology or literacy, with the exception of a significant negative correlation (two-tailed) between duration and PSTM.

**Table 5:** Spearman partial correlations at Time Point 2 showing cross-sectional relations between auditory thresholds and composite scores in literacy and phonological awareness, rapid automatised naming, and phonological short-term memory, controlling for age and NVIQ.

	Sine Rise^†^	SSN Rise^†^	Ba Rise^†^	SSN Intensity^‡^	Ba Duration^‡^	Freq Rise^‡^
PA_TP2	−0.359***	−0.333***	−0.285**	−0.012	−0.178	−0.175
RAN_TP2	0.200*	0.132	0.055	0.010	−0.006	0.010
LIT_TP2	−0.318**	−0.284**	−0.231**	−0.166	−0.136	−0.078
PSTM_TP2	−0.254**	−0.158	−0.105	−0.115	−0.292**	−0.126

CY only (*N* = 88). NVIQ, Non-verbal IQ; PA, Phonological Awareness; RAN, Rapid Automatised Naming: LIT, Literacy. PSTM, phonological short-term memory.

*Correlation significant at 0.05 level († = 1 tailed; ‡ = 2-tailed). **Correlation significant at 0.01 level († = 1 tailed; ‡ = 2-tailed). ***Correlation significant at 0.001 level († = 1 tailed; ‡ = 2-tailed).

To explore whether individual differences in sensitivity to ART indeed predicted later progress in the phonology and literacy measures as expected *a priori*, one-tailed time-lagged partial correlations were computed relating Time Point 1 to Time Point 3 (Table 6, hence approximately ages 8.5–10 years) and also relating Time Point 1 to Time Point 4 (Table 7, hence approximately ages 8.5–11 years). Children with dyslexia who had received an intervention during this period were removed in each case. Inspection of Table 6 shows that all the ART measures were significant longitudinal predictors of individual differences in both phonological awareness and PSTM. The Sine Rise and Ba Rise tasks were also significant longitudinal predictors of RAN, and Ba Rise was a significant longitudinal predictor of literacy. These data support H5, which states that ART sensitivity should show significant relations to phonological development. Regarding the other acoustic measures, the two-tailed time-lagged partial correlations revealed that the frequency rise task was a significant predictor of all four composite measures, showing very similar patterns to Ba Rise. The duration and intensity measures did not show significant longitudinal relations with the phonological measures or the literacy measures, with one exception (duration and phonological awareness).

**Table 6:** Spearman time-lagged correlations between auditory thresholds at Time Point 1 and scores in the composite measures of phonological awareness, RAN, PSTM, and literacy at Time Point 3, controlling for Age and NVIQ (*N* = 58).

	Sine Rise^†^	SSN Rise^†^	Ba Rise^†^	SSN Intensity^‡^	Ba Duration^‡^	Freq Rise^‡^
PA_TP3	−0.255*	−0.291*	−0.456***	−0.256	−0.364**	−0.407**
RAN_TP3	0.310**	0.180	0.340**	0.137	0.212	0.505***
LIT_TP3	−0.197	−0.113	−0.454***	−0.167	−0.254	−0.576***
PSTM_TP3^	−0.365*	−0.263*	−0.397**	−0.176	−0.212	−0.457**

CY only (*N* = 58). NVIQ, Non-verbal IQ; PA, Phonological Awareness; RAN, Rapid Automatised Naming. LIT, Literacy; PSTM, Phonological Short-Term Memory.

^*N* = 43. *Correlation significant at 0.05 level († = 1 tailed; ‡ = 2-tailed). **Correlation significant at 0.01 level († = 1 tailed; ‡ = 2-tailed). ***Correlation significant at 0.001 level († = 1 tailed; ‡ = 2-tailed).

**Table 7:** Spearman time-lagged correlations between auditory thresholds at Time Point 1 and scores in the composite measures of phonological awareness, RAN, PSTM, and literacy at Time Point 4, controlling for age and NVIQ (*N* = 50).

	Sine Rise^†^	SSN Rise^†^	Ba Rise^†^	SSN Intensity^‡^	Ba Duration^‡^	Freq Rise^‡^
PA_TP4	−0.444*	−0.351**	−0.445***	−0.264*	−0.339*	−0.435**
RAN_TP4	0.383**	0.217	0.331*	0.246	0.211	0.337*
LIT_TP4	−0.198	−0.111	−0.419**	−0.314*	−0.376*	−0.515***
PSTM_TP4	−0.345**	−0.195	−0.278*	−0.409**	−0.350*	−0.425**

CY only (*N* = 50). NVIQ, Non-verbal IQ; PA, Phonological Awareness; RAN, Rapid Automatised Naming. LIT, Literacy; PSTM, Phonological Short-Term Memory.

*Correlation significant at 0.05 level († = 1 tailed; ‡ = 2-tailed). **Correlation significant at 0.01 level († = 1 tailed; ‡ = 2-tailed). ***Correlation significant at 0.001 level († = 1 tailed; ‡ = 2-tailed).

Inspection of Table 7 shows that the developmental patterns between auditory sensitivity and phonological development were very similar a year later. All the ART measures were significant predictors of phonological awareness, and both Sine Rise and Ba Rise were significant longitudinal predictors of RAN and PSTM. Ba Rise also predicted literacy 4 years later. Regarding the other acoustic variables, all were significant predictors of phonological awareness, as well as PSTM and literacy. Only RAN showed a less consistent relationship between auditory sensitivity and later performance, whereas only Frequency Rise showed a significant longitudinal relationship.

The consistent longitudinal relations documented in Tables 6, 7 between individual differences in auditory sensitivity and phonological development suggest that auditory processing plays a causal role in the development of phonological awareness. However, the longitudinal analyses conducted so far do not take account of early individual differences in phonological sensitivity, which could also be expected to exert an effect on later phonological awareness. Given the concurrent relations known to be present between auditory sensitivity and phonological processing, this could be sufficient to explain the relations between auditory processing and subsequent phonological skills. To assess this possibility, we also ran a series of step-wise multiple regressions with the phonological awareness composite at Time Point 3 as the dependent variable (DV), taking phonological awareness measured at baseline as the autoregressor (see Table 8). The regressions showed that even after accounting for early phonological sensitivity (which accounted for 22% of unique variance), significant extra variance in later phonological awareness was accounted for by the Ba Rise, Frequency Rise, and Duration measures. The largest unique contributor was Frequency Rise, which accounted for an extra 12% of the unique variance.

**Table 8:** Unique variance (R^2^_change_) in phonological awareness at TP3 explained by the auditory measures after controlling for phonological awareness measured at TP0 as the auto-regressor (*N* = 58).

Step	Beta	R^2^_change_	*p*
1. Age	0.065	0.004	0.63
2. NV_IQ	0.100	0.010	0.46
3. PA TP0	0.469***	0.215	<0.001
4.			
Sine Rise	−0.211	0.038	0.105
SSN Rise	−0.133	0.014	0.319
Ba Rise	−0.283*	0.055	0.048
Freq Rise	−0.357**	0.122	0.003
Intensity	−0.219	0.044	0.079
Ba Duration	−0.262*	0.066	0.031

*p < 0.05, **p < 0.01, ***p < 0.001 (two-tailed).

Finally, it is logically possible that the direction of causality goes both ways and that early phonological sensitivity plays a causal role in subsequent acoustic development. To investigate this possibility, matching analyses to those reported in Table 8 were computed, using the auditory measures as the DV in each case and auditory sensitivity measured at Time Point 1 as the autoregressor. These analyses are reported in the Supplementary Table S4. They did not support the view that phonological sensitivity plays a role in acoustic development. Overall, the longitudinal analyses (which are corrected for age and NVIQ) suggest that the importance of auditory sensory processing for the development of phonological processing skills is a one-way relationship. Phonology is typically considered a cognitive system, but the analyses reported here show that it develops from sensory processing, which itself changes with development. The longitudinal analyses also indicate that the best measure of ART sensitivity changes over development and that sensitivity to rising frequency is comparable to Ba Rise sensitivity in terms of its importance regarding phonology and literacy outcomes.

## 4 Discussion

This is the first longitudinal study to compare the sensitivity of three widely used ART tasks (see Supplementary Table S1) as predictors of children’s development in phonological processing and to also measure the discrimination of duration, intensity, and rising frequency in the same sample of children. Despite the impact of the COVID-19 pandemic on the original longitudinal design, it was still possible to carry out a series of longitudinal analyses. Regarding ART, it was predicted *a priori* that children with dyslexia should show poorer discrimination of ART differences irrespective of the psychoacoustic task used to measure ART (H1). It was also predicted *a priori* that the Ba Rise task might be most sensitive at younger ages given its speech-like structure (H2) and that individual performance as measured by the three different ART tasks should be significantly related (H3), given that different ART tasks should be measuring the same underlying sensory parameter of rise time sensitivity. A Frequency Rise task was also included, as based on Goswami et al. (2013) we expected that frequency rise thresholds would also be elevated in children with dyslexia (H4). Finally, it was predicted that individual differences in ART discrimination should be a significant longitudinal predictor of phonological development (H5) and also of children’s literacy outcomes, which is in line with the prior literature (see Supplementary Table S1). Given the mixed findings in prior developmental studies of other acoustic features such as frequency and duration (e.g., Witton et al., 2019), no *a priori* predictions regarding phonology and literacy were made for the other psychoacoustic tasks.

Regarding H1, the children with dyslexia tested here indeed showed poorer ART discrimination than age-matched control children, but this difference was not consistent across all three ART tasks at the same time point. At the youngest age tested (around 9 years), it was the Ba Rise task that showed significant group differences (see Table 3), consistent with H2. Two years later, the groups were equivalent regarding sensitivity to the Ba Rise stimuli but now differed in ART sensitivity as measured by the Sine Rise and SSN tasks (Table 3). All ART measures were nevertheless significantly related to each other (H3, see Table 4; Figure 3). The exception was the Ba Rise and Sine Rise measures by Time Point 4 (Table 4, age around 11 years). The individual child data visualised in Supplementary Figures S1, S2 showed a wide range of performance in all the ART tasks, whether a child had dyslexia or not. Nevertheless, the children with dyslexia as a group showed slower development in ART sensitivity in all three ART tasks. Overall, H1, H2, and H3 were all supported. Finally, H4 was also supported, as the children with dyslexia consistently showed reduced sensitivity to rising frequency. Indeed, the analyses of internal consistency reliability showed that Frequency Rise loaded onto the same unidimensional internal variable as the ART tasks (Figure 3). This is suggestive of a single acoustic rise time factor.

In terms of relations to phonology (H5), for the cross-sectional analyses carried out at Time Point 2 (Table 5), all the ART measures showed significant relations with phonological awareness, as well as literacy. However, only Sine Rise was related to phonological memory and RAN. In each case, poorer ART discrimination was accompanied by worse performance. Turning to longitudinal relations, the data presented in Table 6 show that the ART measures were significantly related to development in phonological awareness and phonological memory, supporting H5, with two of the three measures (Sine Rise and Ba Rise) also related to progress in RAN. Only Ba Rise was a predictor of literacy. Considering the other psychoacoustic tasks developed for this study (Frequency Rise, Duration, and Intensity), both Frequency Rise and Duration were longitudinal predictors of individual differences in phonological awareness. Otherwise, only the Frequency Rise task showed consistent predictive relations with all outcome measures (phonological awareness, RAN, phonological memory, and literacy, see Table 6). Notably, and in contrast to the ART measures, the concurrent relations between Frequency Rise and these measures were largely non-significant (Table 5).

Similar predictive relations between ART and phonological awareness were found over a longer time period of 3 years (Table 7). The Sine Rise measure continued to predict all the phonological measures (phonological awareness, RAN, and PSTM) but not literacy. The Ba Rise measure continued to be a longitudinal predictor of all the outcome measures, including literacy. Therefore, H5 was broadly supported. The significant longitudinal relationships between auditory processing and later phonological development also survived a stringent control for the effects of earlier phonological sensitivity, as shown by the multiple regression equations using phonological awareness at baseline as the autoregressor (Table 8). Furthermore, the significant longitudinal relationships between auditory processing and later phonological development were not reciprocal, as phonological awareness did not affect later auditory development when earlier auditory sensitivity was included as the autoregressor (Supplementary Table S4). This has also been reported in prior longitudinal ART studies (Goswami et al., 2020). The exception regarding H5 was the SSN Rise task, which only predicted phonological awareness and not literacy. This is surprising given the prior findings for the Dutch, where the SSN Rise task has been a good predictor of literacy (see Supplementary Table S1). However, the roving levels of the SSN standard and target sounds made this a more challenging task for all our participants, as evidenced by floor effects for SSN at Time Point 1 (see Supplementary Figure S1). Regarding the other acoustic measures, all were significant predictors of the composite phonological variables over the longer time span reflected in Table 7. This suggests that multiple aspects of auditory sensitivity are important for the ongoing development of phonological processing. However, only the Frequency Rise task was also a predictor of literacy. Indeed, the relations with the composite variables over 3 years (shown in Table 7) are very similar for both Ba Rise and Frequency Rise, as was also the case over 2 years (shown in Table 6).

As noted, the acoustic structure of the /ba/ stimulus has a different composition to the Sine Rise and SSN stimuli, as it possesses the natural structure of voiced speech. Its fine structure is derived from a harmonic complex tone (HCT). This may explain its highly consistent relations with phonology as well as literacy. HCTs are made up of a fundamental frequency F_0_, and additional frequencies called harmonics. Resonances in the vocal tract boost certain harmonics, resulting in frequencies known as formants, which give rise to individual vowel sounds (Hillenbrand et al., 1995). It is known that subcortical encoding of the higher harmonics in speech tokens (such as the synthetic syllable “da”) is related to the encoding of formant frequencies as well as reading, speech in noise perception, and phonological awareness (Hornickel et al., 2009, 2012; see also Hornickel and Kraus, 2013). The acoustic characteristics of the Frequency Rise task used here were also similar to the dynamic structure of the formants found in speech (Hillenbrand et al., 1995); hence, the task was closer to the natural structure of voiced speech than many previous frequency discrimination tasks. It could also be labelled a spectrotemporal rise time task. This is supported by the latent variable analyses (Figure 3) and could explain the similar patterns regarding the prediction of phonological development and literacy found here for the Ba Rise and Frequency Rise tasks.

The detection of rising frequency is also important for literacy development in Chinese, a tonal language. Regarding phonological development in Chinese, Wang et al. (2019) studied sensitivity to non-speech frequency rise in Chinese–Mandarin speaking children with developmental dyslexia and typical readers (average age 112 months). They reported a significant difference between groups in both lexical tone awareness and sensitivity to frequency sweeps. Lexical tones in Mandarin vary in frequency and frequency contour, typically exhibiting falling or rising frequency. In Wang et al.’s study, Chinese character recognition was significantly correlated with both lexical tone awareness and sensitivity to frequency rise/fall. It is also notable that the Frequency Rise data reported here are consistent with TS theory, which foregrounds the importance of individual differences in sensitivity to *low-frequency modulations* for phonological development. Adult studies have shown that slow FM onsets (3 Hz FM) also phase-reset ongoing cortical oscillations (Henry and Obleser, 2012) in a similar way to slow AM onsets (Gross et al., 2013). A spectrogram of speech reveals not only changes in amplitude over time but concomitant changes in the frequency content. The peripheral auditory system filters the sound signal into an overlapping set of narrowband signals. Accordingly, the filter outputs for a slow frequency sweep can be imagined as a series of separate slow rise times spread across frequency and time, the so-called spectrotemporal rise time. Considering the frequency rise times used here (10–150 ms) as a quarter of a cycle, this equates to modulation rates in the range of 25.0 Hz to 1.7 Hz. The median frequency rise time for all readers (CA, DYS, and RA) was in the region of 119–139 ms, which is equivalent to a frequency modulation rate of 1.8 Hz–2.1 Hz (i.e., 2 Hz has a cycle duration of 500 ms, a quarter cycle = 125 ms). Prior studies relating 2 Hz FM sensitivity in children to individual differences in literacy measures also report significant relations (e.g., Talcott et al., 2002; Boets et al., 2007).

The current study has a number of limitations. The study began just before the COVID-19 pandemic, which had a negative effect on both our ability to collect longitudinal data from the participating children and on their standardised performance in the phonology and literacy tasks, necessitating the use of raw scores for the longitudinal analyses instead of standard scores as used in many prior studies (see Supplementary Table S1). Furthermore, after the pandemic, it was more difficult to access schools, and the task battery had to be reduced; in particular, we could not continue using the Frequency Rise task due to time restrictions. Another limitation is that the study combined longitudinal assessments with aural/oral interventions aimed at improving phonology and literacy outcomes for children with dyslexia. This reduced the number of participants with dyslexia who could contribute data as the longitudinal analyses progressed. A further limitation is that the children were relatively old when the study began, with an average age of 8 years. Although this is fairly typical of the literature reviewed in Supplementary Table S1, given that dyslexia is not usually diagnosed until 3 or 4 years of reading tuition have been provided, TS theory proposes that individual differences in ART sensitivity are present from birth and consequently affect phonological and linguistic development throughout the preschool years, long before reading tuition commences (e.g., Kalashnikova et al., 2019). In future studies, it would be interesting to administer the different acoustic tasks used here to preschool samples and ideally to infants. This would enable a clearer developmental picture of which acoustic features are particularly important for the development of a well-specified phonological system.

In conclusion, the data presented here show evidence of both task effects and age effects regarding relations between both amplitude and frequency rise discrimination and children’s phonology and literacy outcomes. Specifically, for the first time with English-speaking children, greater sensitivity to rising frequency in a sine tone task was shown to be associated with better phonology and literacy outcomes measured months later. Longitudinal relations between greater ART sensitivity and better phonology and literacy outcomes were also documented at all time points for all three ART tasks. Both sets of findings are consistent with TS theory. In particular, the data gathered here suggest that in addition to ART sensitivity, sensitivity to slow-rate spectral modulations is also predictive of outcomes regarding the acquisition of phonology and literacy (Witton et al., 1998; Talcott et al., 2002; Boets et al., 2007).

## Data availability statement

The datasets presented in this article are not readily available because this is an ongoing study and the data are still being analysed by the authors. As the data was obtained from children, our ethics approval does not give us explicit permission to make it publicly available. Some data derivatives will be made available after the completion of the study by contacting the senior author (UG) of the study. Requests to access the datasets should be directed to ucg10@cam.ac.uk.

## Ethics statement

The studies involving humans were approved by the Psychology Research Ethics Committee of the University of Cambridge, UK. The studies were conducted in accordance with the local legislation and institutional requirements. Written informed consent for participation in this study was provided by the participants’ legal guardians/next of kin.

## Author contributions

SF: methodology, investigation, software, data curation, formal analysis, visualisation, and writing—original draft, review, and editing. AW, FG, and AM: investigation and data curation. KM: writing—review & editing. UG: conceptualisation, resources, methodology, investigation, project administration, supervision, funding acquisition, writing – original draft, review, and editing.

## Footnotes

^1^ http://www.neurobs.com/
