# Supplementary material for: Amplitude rise time sensitivity in children with and without dyslexia: differential task effects and longitudinal relations to phonology and literacy
Source: Front Psychol. 2024 Jul 23;15:1245589. doi: 10.3389/fpsyg.2024.1245589 (PMC11302049; doi:10.3389/fpsyg.2024.1245589)
Supplement: Supplementary file 1 [file Table_1.docx]

| Supplementary Table S1.  **Previous Studies utilising Rise Time sensitivity tasks**  Summary table of stimulus used, participant group, thresholds, results.  All Dinosaur 3I-2AFC paradigm unless stated otherwise. Standard = Onset/Steady state/Offset. | | | | |
| --- | --- | --- | --- | --- |
|  |  | | | |
| Study Authors | Method | Group N & Age | Effect Size | Comment |
| Van Herck, S., Economou, M., Vanden Bempt, F., Glatz, T., Ghesquière, P., Vandermosten, M. & Wouters, J., (2023). Neural synchronization and intervention in pre‐readers who later on develop dyslexia. *European Journal of Neuroscience*, *57*(3), pp.547-567. https://doi.org/10.1111/ejn.15894 | ASSR right ear only.  100% SAM noise at 4 Hz and 20 Hz  Pulse noise at syllable (4 Hz) and Phoneme rate (20 Hz) with shortened risetime of 30 ms. | N= 91  5yrs 5 month | No difference found for rise times. | Language: Dutch  Sinusoidal Amplitude Modulated Noise.  Pulse left lateralised, SAM right lateralised in typical readers, only. |
| Van Herck, S., Vanden Bempt, F., Economou, M., Vanderauwera, J., Glatz, T., Dieudonné, B., Vandermosten, M., Ghesquière, P. & Wouters, J., (2022). Ahead of maturation: Enhanced speech envelope training boosts rise time discrimination in pre‐readers at cognitive risk for dyslexia. *Developmental Science*, *25*(3), p.e13186. DOI: 10.1111/desc.13186 | Speech Weighted Noise.  Monaural.  Right ear  Duration 800ms,  RT 15-699ms.  Standard 15/710/75 | N= 91 Pre training  5 – 6 yrs  (6 -7 yrs)  N = 83 post training  N = 79 at +1 yr.  No info on group size changes. | Insufficient detail to calculate effect size. | Language: Dutch  at cognitive risk for dyslexia.  3 groups GGEE/GGNE/ACNE  3 time points  Pre RT= 278/119/157  post RT = 81/92/140  +1yr RT = 84.5/60/55 |
| Keshavarzi et al., (2022)  Decoding of speech information using EEG in children with dyslexia: Less accurate low-frequency representations of speech, not “Noisy” representations*. Brain & Language* 235. https://doi.org/10.1016/j.bandl.2022.105198. | EEG  Sine Tone rise  SSN rise  “Ba” Rise | N=51  30/21  DYS/CA  9.2(0.5)/9.1(0.5) years (SD) | Sine rise d = 0.42  SSN rise d = 0.12  “Ba”Rise d = 0.76 | Language: English  Sine Tone rise = 174/139ms *ns*  SSN rise = 222/215 ms *ns*  “Ba” Rise 101.5/70.8 ms p<.05 |
| Cuetos et al., (2018). Prosodic perception problems in Spanish dyslexia. *Scientific Studies of Reading*, *22*(1), pp.41-54. https://doi.org/10.1080/10888438.2017.1359273 | Sine rise 500 Hz  RT = 15 – 300 ms | N = 96  32/32/32  DYS/CA/RL  7-14 years | RT (*n.s. group diff*)  d = .299  Power < .25  RT: Only CA sig. corr. reading  r = -.399  Syllable stress  r = -.453  rhyme oddity,  r = -.556  Nonword rep.  r = .390  RAN picture  r = .528. | Language: Spanish  Prosodic perception  Wav file number reported.  RT: Only CA sig. correlations with reading, prosody, rhyme oddity, RAN tasks. |
| Chung, W.L., Jarmulowicz, L. and Bidelman, G.M., (2017). Auditory processing, linguistic prosody awareness, and word reading in Mandarin-speaking children learning English. *Reading and Writing*, *30*, pp.1407-1429. DOI:10.1007/s11145-017-9730-8 | Sine rise Tones (as Goswami et al 2013)  Standard 15/735/50 | N = 61  Age 9-10 years | Rise time predicted 10.7% variance in English word reading | Mandarin speakers Taipei. (English instruction from 6yrs)  RT = 122.29 (56.56)  RT sig. correlated with Mandarin final phoneme; English: rhyme, phoneme, word, non-word. |
| Vanvooren, S., Poelmans, H., de Vos, A., Ghesquière, P., & Wouters, J. (2017). Do prereaders’ auditory processing and speech perception predict later literacy? *Research in Developmental Disabilities*, *70*, 138-151. https://doi.org/10.1016/j.ridd.2017.09.005 | Speech Weighted Noise  Monaural Right ear.  RT = 15ms to 699 ms.  800 ms duration  Standard 15/710/75 ms | N=87  44/43  HR/LR  Family risk of DYS  Age 5 years | Risetime correlated with  Phonological awareness –  (1^st^ Grade) 0.31**  Rapid naming –  (1^st^ Grade) 0.34**  Letter Knowledge – (1^st^ Grade) 0.30** Phonological awareness –  (2nd Grade) 0.27*  Rapid naming – (2nd Grade) 0.29** | Repeated measures.  RT performance in kindergarten a predictive factor for phonological awareness at the start of second grade explaining 7.7% ** of the variance. |
| Law, J.M., Vandermosten, M., Ghesquière, P., and Wouters, J. (2017) Predicting Future Reading Problems Based on Pre-reading Auditory Measures: A Longitudinal Study of Children with a Familial Risk of Dyslexia. *Front. Psychol. 8*:124. doi: 10.3389/fpsyg.2017.00124 | Speech Weighted Noise  Monaural Right ear.  RT = 15ms to 699 ms.  800 ms duration  Standard 15/710/75 ms | N = 40  Family Risk of DYS  HR/LR  Age 4-5 years | Kindergarten RT (ms)  Sine rise *d* = 0.77  Grade 1 RT (ms)  Sine rise *d* = 0.70 | English speakers  Retrospective analysis.  RT at 4-5 yrs (Kindergarten) uniquely contributed to growth in Literacy in grade 1** and 2**  Kindergarten RT (ms)  CTL RT = 218 (196.6)  DYS RT = 348 (212.0)  Grade 1 RT (ms)  CTL = 94.0 (59.5)  DYS = 150 (122.0) |
| Study Authors | Method | Group N & Age | Effect Size | Comment |
| Flaugnacco et al (2015) Music Training Increases Phonological Awareness and Reading Skills in Developmental Dyslexia: A Randomized Control Trial. *PLoS ONE, 10,* 9, e0138715. https://doi.org/10.1371/journal.pone.0138715. | Sine 1 kHz,  RT = 10 – 200  800ms duration  Standard 10/780/10 | N= 48  All dyslexic  24/24  Music/AC  Age 8 – 11 years | n/a | Italian Speakers all with dyslexia  Music training intervention effect on rhythm, PA, reading.  No group diff; 83ms before 65 ms after both interventions |
| Beattie, R.L. & Manis, F.R. (2012) Rise Time perception in Children with Reading and combined reading and language Difficulties. *Journal of Learning Disabilities 46*(3) 200–209 | Sine Tone 500 Hz  RT = 15 – 300 ms  Standard 300/450/50  Two rise | N = 50  18/17/15  RD/CA/RD-LD  Age 8 – 16 yrs | Sine Rise  RD/CA *d* = 0.52 | RD- Reading difficulties  RD-LD Reading and oral language difficulties  1 rise no significant difference between CA and RD *p* = .134 |
| Goswami U., Fosker, T., Huss, M. Mead, N., & Szücs, D. (2011).  Rise time and formant transition duration in the discrimination of speech sounds: The Ba-Wa distinction in developmental Dyslexia,  *Developmental Science*, 14 (2011), pp. 34-43, [10.1111/j.1467-7687.2010.00955.x](https://doi.org/10.1111/j.1467-7687.2010.00955.x) | Sine tone  Intensity roved.  RT = 15 – 300 ms  Standard 15/735/50 | N = 106  46/33/27  DYS/CA/RL  DYS/CA age 9yrs 6 months  RL age 7yrs 6 months | Sine rise  CA/DYS *d* = 0.57 | English speakers  CTL RT = 145.2 ms  DYS RT = 193.0 ms  RL RT = 212 ms |
| Poelmans, H., Luts, H., Vandermosten, M., Boets, B., Ghesquiere, P., & Wouters, J. (2011). Reduced sensitivity to slow-rate dynamic auditory information in children with dyslexia*. Research in Developmental Disabilities*, 32(6), 2810–2819. https://doi.org/10.1016/j.ridd.2011.05. 025 | Speech weighted noise  Monaurally  RT 15-300  Standard 15/710/75 | N=58  25/20/13  LR/HR/DYS  Age 11 yrs | Rise time CTL/DYS  *d* = 0.64 | Dutch speakers  Risk of dyslexia and controls  CTL RT = 70.1(50.1) ms  DYS RT = 104.0 (61.8) ms (p=.019) |
| Huss, M., Verney, J., Fosker, T., Mead, N. & Goswami U., (2011) Music rhythm, rise time perception and developmental dyslexia: Perception of musical meter predicts reading and phonology. *Cortex* 47 674-689 | Sine Tone 500 Hz  One Rise 15-300 ms  Standard 15/735/50  Rise Duration Rove  Steady state 450-735 | N = 64  DYS/CA/RL  33/16/15  Age 10.5/10.5/8.33 yrs | One Rise  *d* = 1.14  One Rise time rove  *d* = 1.29 | English speakers  Rise time  CTL RT = 36.4 (14.8) ms  DYS RT 105.9 (73.0) ms  Rise Duration Rove  CTL RT = 31.8 (11.7) ms  DYS RT 113.4 (76.3) ms |
| Study Authors | Method | Group N & Age | Effect Size | Comment |
| Wang H-L. S., Huss, M., Hämäläinen, J.A. and Goswami, U. (2010 online) *Read Writ* (2012) 25:509–536 DOI 10.1007/s11145-010-9284-5 | Sine Tone 500 Hz  One Rise 15-300 ms  Standard 15/735/50 ms | N= 73  26/29/18  DYS/CA/RL  Age 10/8.5 yrs | Sine one rise  *d* = 0.88  Sine two rise  *d* = 0.48 | Native Mandarin  One rise DYS>CA  *F*(2,70) = 4.36*  Two rise *ns* |
| Goswami, U., Wang, H. L., Cruz, A., Fosker, T., Mead, N., & Huss, M. (2011). Language-universal sensory deficits in developmental dyslexia: English, Spanish, and Chinese. *Journal of cognitive neuroscience*, *23*(2), 325–337. https://doi.org/10.1162/jocn.2010.21453 | Sine Tone 500 Hz  One Rise 15-300 ms  Standard 15/735/50 ms | N = 71  44/27  DYS/CA  Age 10.5 yrs | Sine one rise  English *d* =1.14  Spanish *d* = 0.87 | English  CA RT = 36.5 (14.6)  DYS RT = 109.6 (80.4)  Spanish  CA RT = 73.0 (43.8)  DYS RT = 124.2 (73.1) |
| Fraser, J., Goswami, U., Conti-Ramsden, G. (2010) Dyslexia and Specific Language Impairment: The Role of Phonology and Auditory Processing, *Scientific Studies of Reading, 14*(1), 8-29, DOI: 10.1080/10888430903242068 | Sine Tone 500 Hz  One rise AXB  Standard 300/700/50  Two rise 2IFC  RT 15 -300  Standard 300 ms | N = 64  DYS/SLI/DYS+SLI/CA  14/16/21/13 | DYS/CA  One Rise *d* = 0.91  Two rise *d* = 1.29 | n.s. group difference |
| Hämäläinen, et al., (2009).  Common variance in amplitude envelope perception tasks and their impact on phoneme duration perception and reading and spelling in Finnish children with reading. Applied Psycholinguistics, 30, 511–530. http://dx.doi.org/10.1017/ S0142716409090250 | Sine tone 500 Hz  Standard 300/700/50 | N=60  30/30  DYS/CA  Age 9.0 (0.4) years | One rise *d* = 0.2 | Language: Finnish |
| Richardson, U., Thomson, J. M., Scott, S. K., & Goswami, U. (2004). Auditory processing skills and phonological representation in dyslexic children. *Dyslexia, 10*(3), 215–233. https://doi.org/10.1002/dys.276 | Sine tone 500 Hz  One rise AXB  RT = 15 – 300 ms  Standard 15/700/50  Target RT/700/50 hence varied in length 765 – 1050 ms.  2 rise 2IFC  Modulation RT 15 – 300 ms  Standard 300 | N = 65  24/24/17  DYS/CA/RL  Age years, months  8,9/8,10/7,3 | One Rise *d* = 0.75  Two Rise *d* = 0.72 | One rise: target rise time also varies the duration which is a potential confound.  2 Rise. The onset is the same in both conditions with a modulation in the stimulus. The target modulation rise time varies from 300 to 15 ms. The child is asked to choose the “clearer beat”. |
| Note: DYS = Dyslexic; RD = Reading Difficulties; CA = Chronological age matched control; RL = Reading level matched control; SLI = Specific Language Impairment; LIQPR = Low IQ Poor Reader; RT = Rise Time; ASSR = Auditory Steady-State Response; SAM = Sinusoidal Amplitude Modulated; GG = GraphoGame; EE = envelope enhanced; NE = No Enhancement; AC = Active Control; HR = High Risk; LR = Low Risk. **p< .01, *p< .05 | | | | |
|  | | | | |

References

Beattie, R., and Manis, F. (2012). Rise Time Perception in Children with Reading and Combined Reading and Language Difficulties. *Journal of Learning Disabilities*, *46*(3), 200-209. https://doi.org/10.1177/0022219412449421

Chung, W.L., Jarmulowicz, L. and Bidelman, G.M., (2017). Auditory processing, linguistic prosody awareness, and word reading in Mandarin-speaking children learning English. *Reading and Writing*, *30*, pp.1407-1429. DOI:10.1007/s11145-017-9730-8

Cuetos, F., Martínez-García, C. and Suárez-Coalla, P., (2018). Prosodic perception problems in Spanish dyslexia. *Scientific Studies of Reading*, *22*(1), pp.41-54. https://doi.org/10.1080/10888438.2017.1359273

Flaugnacco, E., Lopez, L., Terribili, C., Montico, M., Zoia, S., and Schön, D. (2015). Music Training Increases Phonological Awareness and Reading Skills in Developmental Dyslexia: A Randomized Control Trial. *PLoS ONE, 10,* 9, e0138715. <https://doi.org/10.1371/journal.pone.0138715>.

Fraser, J., Goswami, U., and Conti-Ramsden, G. (2010). Dyslexia and specific language impairment: The role of phonology and auditory processing. *Scientific Studies of Reading*, *14*, 8-29. [https://doi.org/10.1080/10888430903242068](https://doi/10.1080/10888430903242068)

Goswami U., Fosker, T., Huss, M. Mead, N., & Szücs, D. (2011). Rise time and formant transition duration in the discrimination of speech sounds: The Ba-Wa distinction in developmental Dyslexia. ,Developmental Science, 14,.34-43, [10.1111/j.1467-7687.2010.00955.x](https://doi.org/10.1111/j.1467-7687.2010.00955.x)

Goswami, U., Wang, H. L., Cruz, A., Fosker, T., Mead, N., & Huss, M. (2011). Language-universal sensory deficits in developmental dyslexia: English, Spanish, and Chinese. *Journal of cognitive neuroscience*, *23*(2), 325–337. <https://doi.org/10.1162/jocn.2010.21453>

Hämäläinen, J. A., Leppänen, P. H. T., Eklund, K., Thomson, J. M., Richardson, U., Guttorm, T. K., Witton, C., Poikkeus, A-M., Goswami, U., & Lyytinen, H. (2009). Common variance in amplitude envelope perception tasks and their impact on phoneme duration perception and reading and spelling in Finnish children with reading disabilities. Applied Psycholinguistics, 30(3), 511-530. [https://doi.org/10.1017/S0142716409090250](https://doi/10.1017/S0142716409090250)

Huss, M., Verney, J., Fosker, T., Mead, N. & Goswami U., (2011) Music rhythm, rise time perception and developmental dyslexia: Perception of musical meter predicts reading and phonology. *Cortex* 47 674-689

Keshavarzi, M., Mandke, K., Macfarlane, A., Parvez, L., Gabrielczyk, F., Wilson, A., Flanagan, S., Goswami, U. (2022). Decoding of speech information using EEG in children with dyslexia: Less accurate low-frequency representations of speech, not "Noisy" representations. Brain and Language, 235, 105198, ISSN 0093-934X. [https://doi.org/10.1016/j.bandl.2022.105198.](https://www.sciencedirect.com/science/article/pii/S0093934X22001286)

Law, J.M., Vandermosten, M., Ghesquière, P., and Wouters, J. (2017) Predicting Future Reading Problems Based on Pre-reading Auditory Measures: A Longitudinal Study of Children with a Familial Risk of Dyslexia. *Front. Psychol. 8*:124. doi: 10.3389/fpsyg.2017.00124.

Poelmans, H., Luts, H., Vandermosten, M., Boets, B., Ghesquiere, P., & Wouters, J. (2011). Reduced sensitivity to slow-rate dynamic auditory information in children with dyslexia*. Research in Developmental Disabilities*, 32(6), 2810–2819. https://doi.org/10.1016/j.ridd.2011.05. 025

Richardson, U., Thomson, J. M., Scott, S. K., & Goswami, U. (2004). Auditory processing skills and phonological representation in dyslexic children. *Dyslexia, 10*(3), 215–233. https://doi.org/10.1002/dys.276

Van Herck, S., Economou, M., Vanden Bempt, F., Glatz, T., Ghesquière, P., Vandermosten, M. and Wouters, J., (2023). Neural synchronization and intervention in pre‐readers who later on develop dyslexia. *European Journal of Neuroscience*, *57*(3), pp.547-567. https://doi.org/10.1111/ejn.15894

Van Herck, S., Vanden Bempt, F., Economou, M., Vanderauwera, J., Glatz, T., Dieudonné, B., Vandermosten, M., Ghesquière, P. and Wouters, J., (2022). Ahead of maturation: Enhanced speech envelope training boosts rise time discrimination in pre‐readers at cognitive risk for dyslexia. *Developmental Science*, *25*(3), p.e13186. DOI: 10.1111/desc.13186

Vanvooren, S., Poelmans, H., de Vos, A., Ghesquière, P., & Wouters, J. (2017). Do prereaders’ auditory processing and speech perception predict later literacy? *Research in Developmental Disabilities*, *70*, 138-151. https://doi.org/10.1016/j.ridd.2017.09.005

Wang H-L. S., Huss, M., Hämäläinen, J.A. and Goswami, U. (2010 online) *Read Writ* (2012) 25:509–536. DOI 10.1007/s11145-010-9284-5
