# Supplementary material for: Amplitude rise time sensitivity in children with and without dyslexia: differential task effects and longitudinal relations to phonology and literacy
Source: Front Psychol. 2024 Jul 23;15:1245589. doi: 10.3389/fpsyg.2024.1245589 (PMC11302049; doi:10.3389/fpsyg.2024.1245589)
Supplement: Supplementary file 2 [file Data_Sheet_1.docx]

| **Supplementary Table 2**  Participant chronological age and reading age by group.  Median values in months. | | | | |
| --- | --- | --- | --- | --- |
| **Time Point 2** | CA (*n* = 30) | DYS (*n* = 58) | RA (*n* = 32) | H(2) |
| Chronological Age^a^ | 108 | 112 | 91.5 | 60.38*** |
| Reading Age ^b^ | 111 | 85 | 89.5 | 55.98*** |
| *Difference* | *+3* | *-27* | *-2* |  |
|  |  |  |  |  |
| **Time Point 3** | CA (*n* = 25) | DYS (*n* = 50) | RA (*n* = 18) | H(2) |
| Chronological Age^a^ | 120 | 123 | 104 | 35.44*** |
| Reading Age ^b^ | 117 | 91 | 105 | 35.47*** |
| *Difference* | *-3* | *-32* | *+1* |  |
|  |  |  |  |  |
| **Time point 4** | CA (*n* = 29) | DYS (*n* = 49) | RA (*n* = 29) | H(2) |
| Chronological Age^a^ | 131 | 134.5 | 115 | 53.36*** |
| Reading Age ^c^ | 129 | 99 | 117 | 35.83*** |
| *Difference* | *-2* | *-35.5* | *+2* |  |

Note:

^a^ Kruskal-Wallis test. CA = DYS> RA. Bonferroni Adjusted for multiple tests.

^b^ Kruskal-Wallis test. CA > DYS = RA. Bonferroni Adjusted for multiple tests.

^c^ Kruskal-Wallis test. CA > RA> DYS. Bonferroni Adjusted for multiple tests.

****p* < .001

| **Supplementary Table 3**  Simple linear regression of age on auditory thresholds for the 63 typical readers (CA and RA). | | | | |
| --- | --- | --- | --- | --- |
|  | Beta | R^2^ | F(1,62) | p |
| **Time Point 1** |  |  |  |  |
| Sine rise | -.369 | .136 | 9.61 | .003 |
| Ba rise | -.496 | .246 | 19.91 | <.001 |
| Frequency rise | -472 | .223 | 17.46 | <.001 |
| Duration | -.414 | .171 | 12.62 | <.001 |
| Intensity | -.359 | .129 | 9.04 | .004 |
| **Time Point 2** |  |  | F(1,61) |  |
| SSN | -.318 | .101 | 6.73 | .012 |

S3. Results

S3.1 Differential Task Effects

To enable further visualisation of the developmental differences, Figures S1 and S2 provide box plots comparing thresholds for Time Point 1 versus Time Point 3 for all 6 psychoacoustic measures (those DYS children receiving the first oral intervention are removed at Time Point 3). As will be recalled, due to the Pandemic Time Point 3 was the last time point that all six psychoacoustic tasks could be administered. Inspection of Figure S1 is consistent with H2, that the Ba Rise measure would be the most sensitive ART measure developmentally. Both typically-developing groups appear to achieve lower thresholds than the DYS group by Time Point 3, although some younger RA children still showed relatively poor sensitivity. Figure S2 shows a similar pattern of slower development for the children with dyslexia for sensitivity to Frequency Rise and Duration, although for the younger reading-age matched children the distribution for Frequency Rise appears bimodal. Notably, however, the DYS group was not impaired in discriminating intensity at either time point. Overall, the data depicted in Figures S1and S2 indicate that all groups were developing in terms of acoustic sensitivity, but that the children with dyslexia showed slower development, particularly regarding the ART tasks (consistent with H1).


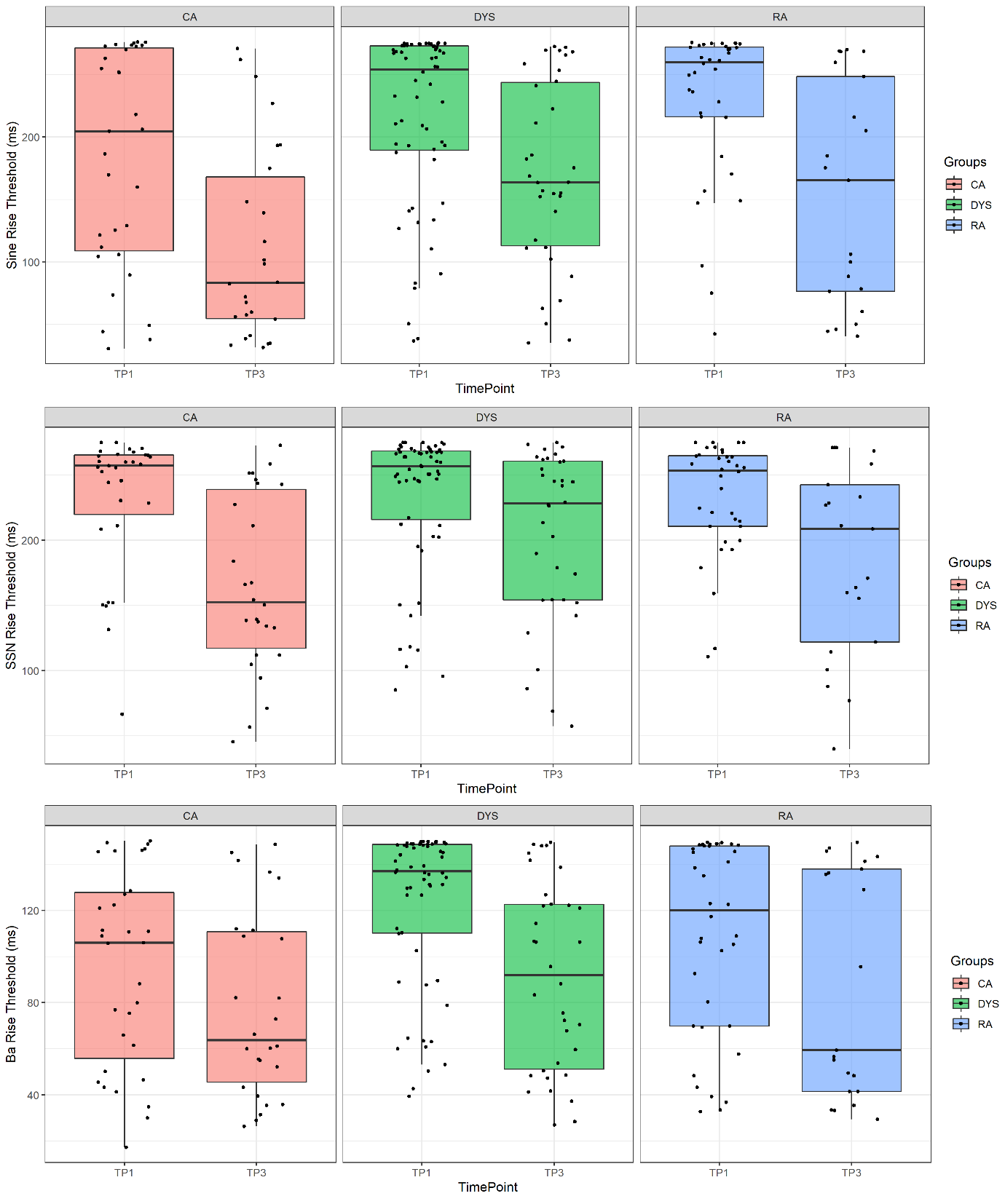


Supplementary Figure S1.

**Cross sectional analysis.** Measures for groups DYS (without intervention) and CA were included for the cross-sectional analysis. Boxplots of ART sensitivity by task at Time Points 1 and 3. Bold line represents the Median. Note different ordinate scale for Ba rise.


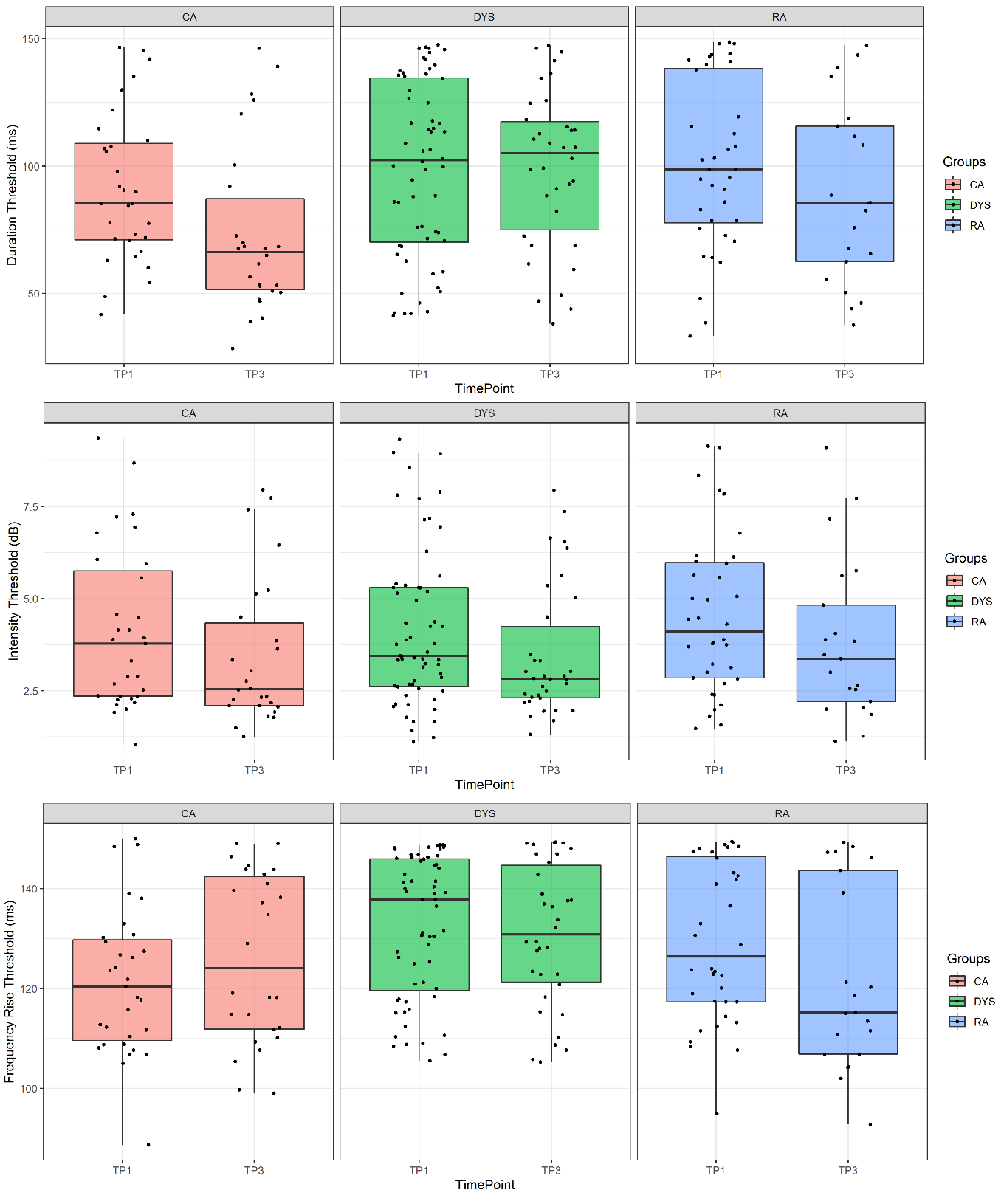
Supplementary Figure S2.

**Cross sectional analysis.** Measures for groups DYS (without intervention) and CA were included for the cross-sectional analysis. Boxplots of Duration, Intensity and Frequency rise thresholds by task at Time Points 1 and 3. Bold line represents the Median.

S3.2 Phonological Sensitivity and Auditory Development

It is conceivable that the direction of causality goes both ways, and that early phonological sensitivity plays a causal role in subsequent acoustic development. This possibility was explored by running a second series of step-wise multiple regressions, this time taking each of the three auditory measures that were significant in Table 8 at Timepoint 3 as the DV (Ba Rise, Frequency Rise, duration), and using auditory sensitivity measured at Time Point 1 as the autoregressor (see Table S4). The data are not supportive of a reverse causal relationship. Early phonological skills do not appear to be causally related to developments in auditory processing. For each auditory variable, the autoregressor was significant, and early phonological sensitivity failed to account for any further unique variance in auditory processing measured at Time Point 3.

| **Supplementary Table 4.**  **Regression Equations assessing the Longitudinal Influence of Phonological Awareness on Auditory Processing**  Unique Variance (R^2^_change_ ) in Freq Rise at TP3 explained by Phonological Awareness measured at TP0 with Freq Rise at TP1 as the Auto-regressor **(**N =58) | | | | | |
| --- | --- | --- | --- | --- | --- |
| **Step** | **Beta** | **R^2^_change_** | ***p*** |  |  |
| 1. **Age** | -.400 | .160 | .002 |  |  |
| 1. **NV_IQ** | -.107 | .011 | .391 |  |  |
| 1. **F**req **Rise TP1** | .372 | .137 | .002 |  |  |
| 1. **PA TP0** | .032 | .001 | .786 |  |  |
| 1. Unique Variance (R^2^_change_ ) in Ba_Duration at TP3 explained by Phonological Awareness measured at TP0 with Ba_Duration at TP1 as the Auto-regressor (N=58) | | | | |  |
| 1. **Age** | -.005 | .000 | .971 |  |  |
| 1. **NV_IQ** | -.207 | .042 | .124 |  |  |
| 1. **Ba_Dur TP1** | .377 | .139 | .004 |  |  |
| 1. **PA TP0** | -.053 | .003 | .681 |  |  |
| **(c)** Unique Variance (R^2^_change_ ) in Ba_Rise at TP3 explained by Phonological Awareness measured at TP0 with Ba_Rise at TP1 as the Auto-regressor (N =58) | | | | |  |
| **Step** | Beta | **R^2^_change_** | ***p*** |  |  |
| 1. **Age** | -.256 | .066 | .052 |  |  |
| **2. NV_IQ** | -.077 | .006 | .558 |  |  |
| **3.Ba Rise TP1** | .507 | .241 | <.001 |  |  |
| **4.PA TP0** | -.046 | .002 | .733 |  |  |

| Supplementary Table 5.  Factor loadings, R^2^, and error variances for single factor model using Maximum Likelihood estimation. | | | |
| --- | --- | --- | --- |
| TP1 | Standardized loadings | R^2^ | Error Variance |
| SSN | 0.54 | 0.29 | 0.71 |
| Sine | 0.64 | 0.41 | 0.59 |
| Ba | 0.70 | 0.49 | 0.51 |
| F0 | 0.54 | 0.30 | 0.70 |
|  |  |  |  |
| TP2 | Standardized loadings | R^2^ | Error Variance |
| SSN | 0.50 | 0.25 | 0.75 |
| Sine | 0.78 | 0.61 | 0.39 |
| Ba | 0.57 | 0.32 | 0.68 |
| F0 | 0.71 | 0.50 | 0.50 |
|  |  |  |  |
| TP3 | Standardized loadings | R^2^ | Error Variance |
| SSN | 0.63 | 0.40 | 0.60 |
| Sine | 0.77 | 0.59 | 0.41 |
| Ba | 0.64 | 0.41 | 0.59 |
| F0 | 0.59 | 0.34 | 0.66 |
